# MreB-Dependent Inhibition of Cell Elongation during the Escape from Competence in *Bacillus subtilis*

**DOI:** 10.1371/journal.pgen.1005299

**Published:** 2015-06-19

**Authors:** Nicolas Mirouze, Cécile Ferret, Zhizhong Yao, Arnaud Chastanet, Rut Carballido-López

**Affiliations:** 1 INRA, UMR 1319 Micalis, Jouy-en-Josas, France; 2 AgroParisTech, UMR 1319 Micalis, Jouy-en-Josas, France; Max Planck Institute for Terrestrial Microbiology, GERMANY

## Abstract

During bacterial exponential growth, the morphogenetic actin-like MreB proteins form membrane-associated assemblies that move processively following trajectories perpendicular to the long axis of the cell. Such MreB structures are thought to scaffold and restrict the movement of peptidoglycan synthesizing machineries, thereby coordinating sidewall elongation. In *Bacillus subtilis*, this function is performed by the redundant action of three MreB isoforms, namely MreB, Mbl and MreBH. *mreB* and *mbl* are highly transcribed from vegetative promoters. We have found that their expression is maximal at the end of exponential phase, and rapidly decreases to a low basal level upon entering stationary phase. However, in cells developing genetic competence, a stationary phase physiological adaptation, expression of *mreB* was specifically reactivated by the central competence regulator ComK. In competent cells, MreB was found in complex with several competence proteins by *in vitro* pull-down assays. In addition, it co-localized with the polar clusters formed by the late competence peripheral protein ComGA, in a ComGA-dependent manner. ComGA has been shown to be essential for the inhibition of cell elongation characteristic of cells escaping the competence state. We show here that the pathway controlling this elongation inhibition also involves MreB. Our findings suggest that ComGA sequesters MreB to prevent cell elongation and therefore the escape from competence.

## Author Summary

In bacterial cells, like in their eukaryotic counterparts, precise spatiotemporal localization of proteins is critical for their cellular function. This study shows that the expression and the localization of the bacterial actin-like MreB protein are growth phase-dependent. During exponential growth, we previously showed that MreB, together with other morphogenetic factors, forms discrete assemblies that move in a directed manner along peripheral tracks. Here, we demonstrate that in cells that develop genetic competence during stationary phase, transcription of *mreB* is specifically activated and MreB relocalizes to the cell poles. Our findings suggest a model in which MreB sequestration by the late competence protein ComGA prevents cell elongation during the escape from competence.

## Introduction

In response to nutritional deprivation and high population density, the rod-shaped model Gram-positive bacterium *Bacillus subtilis* enters stationary phase and develops diverse environmental adaptations, namely competence for genetic transformation, sporulation, cannibalism or biofilm formation [1]. These developmental programs are exquisitely regulated in order to anticipate starvation and optimize the survival of at least a fraction of the population. During the development of these adaptations, cells initiate a large reorganization of gene expression [2,3], protein localization [4,5] and cell shape [5].

In the case of genetic competence, the central regulator ComK activates the expression of more than a hundred genes [2,6,7]. Competence development in *B*. *subtilis* is a well-known bistable system [1]. Only a small fraction of a population (2 to 10%) expresses the ComK-dependent genes, and thus the large majority of the population remains in the non-competent state [8,9]. Within the ComK regulon, twenty-eight genes are essential for genetic transformation [10], a process defined as the genetic alteration of a competent cell by incorporation of foreign DNA in its genome. The remaining genes upregulated in the presence of ComK may be involved in functions other than transformation. Accordingly, it was proposed to rename the ComK-determined physiological state the K-state, a more neutral term than genetic competence [2]. For instance, it has been shown that growth is inhibited during the escape from competence. When the environmental conditions improve (e.g. upon dilution into fresh medium), non-competent cells rapidly resume growth whereas competent cells remain in a growth-limited state during which both cell elongation and cell division remain inhibited for more than 90 minutes before they start to grow again [11,12]. This delay relative to non-competent cells is thought to constitute a tightly regulated checkpoint to allow the repair of the chromosome following homologous recombination of the transforming DNA, before replication initiation [11,12]. Growth inhibition during the escape from competence is controlled at two levels: cell elongation is inhibited through the late competence peripheral protein ComGA [11] and cell division is inhibited by ComGA and the highly conserved protein Maf [11,12]. The ComGA-mediated mechanism that inhibits cell elongation during outgrowth remains unknown. After exhibiting a diffuse localization in the cytoplasm, ComGA accumulates preferentially at polar clusters where it co-localizes with other competence proteins to form the transformation machinery [4,13]. Upon dilution into fresh medium, ComGA stays at the poles for 120 minutes before delocalizing, presumably through degradation or inactivation, ultimately reversing elongation inhibition [12].

Among the different classes of proteins regulating bacterial cell elongation, the bacterial actin-like MreB proteins have been the most studied over the past fifteen years. MreB proteins (Mre, for Murein cluster e) are essential for cell morphogenesis in most non-spherical bacteria [14,15]. In exponentially growing rod-shaped cells, MreB proteins localize in membrane-associated assemblies that rotate perpendicularly to the long axis of the cell [16–21]. These MreB structures are thought to control cell elongation by directing the assembly and movement of macromolecular complexes that effect synthesis of the sidewalls (cell cylinder) during growth [14,16,17].

In *B*. *subtilis*, sidewall elongation during vegetative growth is controlled by the redundant action of three MreB isoforms: MreB, Mbl and MreBH [22]. *mreB* and *mbl* are essential under normal growth conditions [23,24], while *mreBH* is essential only under certain adverse conditions [22,25]. The *mreB* gene is found in the third position of an operon composed of seven genes; immediately upstream the *mreCD* morphogenes and the *minCD* division-related genes, and downstream *maf*, involved in division inhibition during competence [12], and *radC*, of yet unknown function. It has been shown that several promoters are located within or upstream the *mreB* operon [12,26,27]. *mbl* is found immediately downstream *spoIIID*, a gene encoding a sporulation-specific transcriptional regulator [28] and *usd*, a gene located upstream *spoIIID* and necessary for its translation [29]. A sigma-E dependent promoter, activating the *mbl* expression during sporulation, is located upstream *usd* and *spoIIID* [26,30]. However, it has been shown that expression of *mbl* during vegetative growth is ensured by a sigma-A dependent promoter located between *spoIIID* and *mbl* [26]. Finally, *mreBH* forms an operon with a small gene of unknown function, *ykpC* [26]. Transcription of the *mreBH* operon is driven by the alternative sigma factor sigma-I, which is induced during heat shock [31]. The specific expression of the three *mreB* isoforms, from different promoters depending on different sigma factors, is in agreement with their partial functional redundancy upon various stress conditions [22].

Interestingly, *mreB* and *mbl* were identified as competence-induced genes in a transcriptomic study [2]. However, a detailed profile of expression of these two genes throughout growth and stationary phase remained to be characterized, and a possible role of MreB-like proteins in stationary phase adaptations was not investigated so far. Here, we report a new role associated to MreB during genetic competence in *B*. *subtilis*. We show that *mreB* (but not *mbl*) belongs to the ComK regulon, and that in competent cells MreB forms a complex with several competence proteins. Additionally, MreB co-localizes with ComGA in polar clusters. We finally show that ComGA-dependent growth inhibition displayed by cells escaping the K-state also involves MreB. We propose a model in which ComGA sequesters MreB in order to prevent cell elongation during outgrowth and therefore the escape from competence.

## Results

### *mreB* is a competence-induced gene, regulated by ComK

In previous transcriptional profiling studies of *B*. *subtilis* grown to competence, all the genes of the *mreB* operon were found to be down-regulated in *comK* mutant cells relative to wild-type cells [2]. *mbl* was also down-regulated but only when *mecA*, which codes for the adaptor protein that targets ComK for proteolysis [32], was knocked-out to increase the percentage of competent cells [33]. It was proposed that expression of both *mreB* and *mbl* was ComK-dependent and thus induced during competence, although it could not be excluded that *mbl* expression was affected by ComK only in the pleiotropic *mecA* background [2].

**Fig 1:**
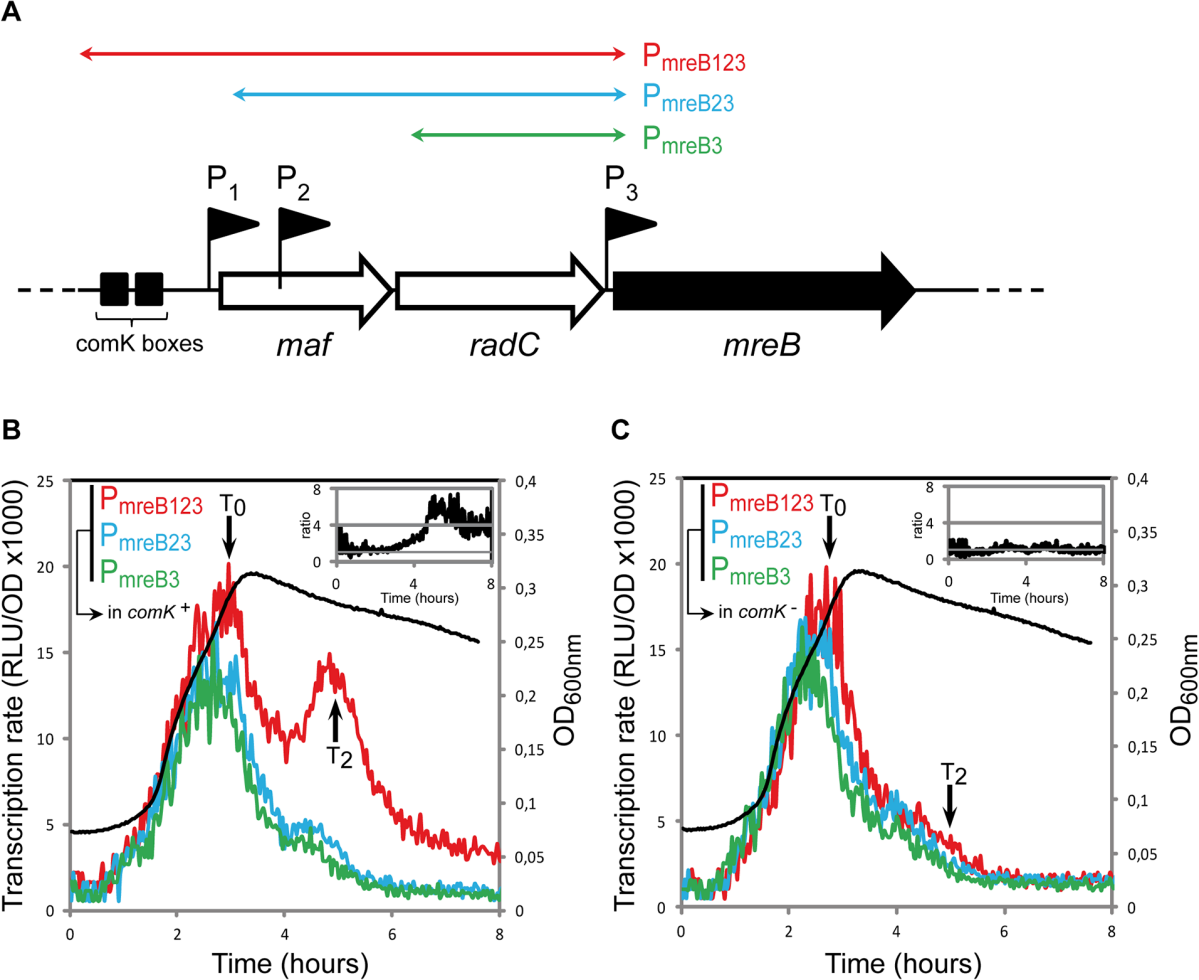
*mreB* is a competence-regulated gene. A. Partial map of the *mreB* operon. The three first genes of the operon, *maf*, *radC* and *mreB* are displayed. The three promoter’s upstream *mreB* (P1, P2 and P3) are represented by black flags. The two black boxes in front of the operon indicate the ComK binding sites that allow competence-specific over-expression of the operon [12]. The three colored double-headed arrows delimit the fragments used in the luciferase assay to characterize the expression coming from each promoter. P_*mreB123*_ (in red, strain NC91) contains the three promoters and the ComK boxes, P_*mreB23*_ (in blue, NC92) contains promoters P2 and P3, and P_*mreB3*_ (in green, NC93) only contains the last promoter in front of *mreB*, P3. B. Transcription profiles of strains expressing P_*mreB123*_ *-luc* (in red, NC91), P_*mreB23*_ *-luc* (in blue, NC92) or P_*mreB3*_ *-luc* (in green, NC93) in a *comK*+ background during growth in competence medium at 37°C. Transcription rates are presented as the evolution of relative luminescence units corrected for OD (RLU/OD, left y-axis). As a reference, the NC91 growth curve (in black) is represented (OD_*600nm*_, right y-axis). The black arrows denote time points (in hours) relative to the beginning of competence (T_*0*_). The three cultures grew almost identically (see S1A Fig) The inset on the top right corner shows the evolution of the ratio between the expression detected from P_*mreB123*_ and P_*mreB3*_. C. Same as B except that transcription profiles were measured in a *comK* mutant background (in red, strain NC146; in blue, NC147; in green, NC148). As a reference, the NC146 growth curve (in black) is represented (OD_*600nm*_, right y-axis). The three cultures grew almost identically (see S1B Fig). The inset on the top right corner shows the evolution of the ratio between the expression detected from P_*mreB123*_ and P_*mreB3*_ during time in the absence of *comK*.

We examined whether transcription of *mreB*, *mbl* and/or *mreBH* was specifically induced during competence. Fragments of different sizes (500 to 2300 bp) upstream the open-reading frames of *mreB* (Fig 1A), *mbl* (S2A Fig) *and mreBH* (S2B Fig) containing several promoters were fused to the firefly luciferase (*luc*) coding sequence. In the case of *mreB*, three promoters were previously identified: P1, upstream the *maf-radC-mreBCD-minCD* operon [12,26]; P2, inside *maf* [27] and P3, between *radC* and *mreB* [26,27] (Fig 1A). P1 and P3 are dependent on the major housekeeping sigma factor sigma-A, while P2 is dependent on extracytoplasmic sigma factors [26,27,34]. P1 also contains ComK binding boxes (Fig 1A) and was shown to drive expression of *maf* during competence [12]. We measured the transcription rate from three fragments upstream *mreB*: P_mreB123,_ containing the three promoters; P_mreB23,_ containing promoters P2 and P3, and P_mreB3,_ containing P3 (Fig 1A) during growth (measured by OD_600,_ S1 Fig) in competence medium (CM). During exponential growth, expression of *luc* fused to P_mreB123_, P_mreB23_ and P_mreB3_ was virtually identical (Fig 1B). The transcription rate progressively increased to reach a maximum during the transition from exponential growth to stationary phase, which marks the beginning of competence (T_0_). This indicated that in exponentially growing cells expression of *mreB* comes from P3. No transcript generated from P1 and P2 could be detected, even if P1 has been shown to drive the low, basal expression of *maf* during exponential growth [12]. Upon entering stationary phase (after T_0_), the transcription rate from all three fragments rapidly decreased (Fig 1B). Transcription from P_mreB23_ (P2 + P3) and P_mreB3_ (P3 alone) exhibited a relatively sharp and progressive decrease, reaching a low basal level approximately 3 h after T_0_. However, expression from P_mreB123_ (P1 + P2 + P3) was significantly higher and exhibited a prominent burst about 2 hours after T_0_ (T_2_, which corresponds to the time of maximal competence [35]). Thus, in stationary phase there was a substantial (4–6 fold, see inset in Fig 1B) increase in *mreB* transcription that came from P1, the promoter in front of the operon. This was consistent with the recent finding that *maf* is expressed during competence from P1, regulated by the master regulator ComK [12]. As expected, when we monitored *mreB* transcription in a *comK* mutant, the transcriptional burst observed at T_2_ was abolished and the transcription rate from P_mreB123_ was comparable to that from the two shorter fragments P_mreB23_ and P_mreB3_ (Fig 1C).

Two promoters were previously identified for *mbl*: P1, a sigma-E-dependent promoter located upstream the *usd* gene and P2, a sigma-A-dependent promoter right upstream *mbl* (S2A Fig) [26,30,36]. Like *mreB*, *mbl* was transcribed predominantly during exponential growth, and maximum of expression was reached right before T_0_ (S2C Fig). Expression of *mbl* in exponentially growing cells came exclusively from P2. In contrast to *mreB*, however, expression of *mbl* was not reactivated in stationary phase and was not affected by ComK (S2C Fig), even though a small peak can be observed around T_2_. *mbl* was previously reported to be over-expressed in *comK* mutant cells only when *mecA* was also knocked-out [2]. Since *mecA* mutants are very pleiotropic [37,38], our results indicate that activation of *mbl* transcription in the *comK* ^-^ *mecA* ^-^ background was indirect, resulting from secondary effects of the absence of *mecA*. For *mreBH*, only a sigma-I-dependent promoter, induced during heat shock, has been identified [31]. Consistently, no transcription of *mreBH* was detected during growth in competence medium (S2D Fig).

Taken together, our findings indicate that *mreB*, but not *mbl* and *mreBH*, is a competence-induced gene, regulated by ComK.

### MreB is associated to ComGA in competent cells

To provide insight into a possible role of MreB in competent cells, we sought to identify MreB binding partners during competence. To this end, MreB was fused to the sequential peptide affinity (SPA) tag [39]. Unlike cells lacking MreB, cells containing *spa*-*mreB* as only copy of *mreB* in their genome displayed normal morphology in both exponential and stationary phase (S3 Fig) indicating that the SPA-MreB fusion was functional. The strain expressing the SPA-MreB fusion was grown to T_2_ in CM at 37°C, and MreB-associated proteins were purified and identified by mass spectrometry. Strains expressing no SPA-tagged protein and a SPA fusion to PerR, a non-related protein of *B*. *subtilis*, were used as negative controls. Interestingly, several competence proteins (ComGA, Maf, ComEB, ComC and ComFA) were specifically and reproductively detected in the MreB pull-down complexes (Table 1). Among these proteins, ComGA was the most abundant in the complex based on the Protein Abundance Index (PAI, established according to [40]). ComGA was co-purified with SPA-MreB well above the contaminant value found in the control strains (Table 1), indicating that their co-purification was specific. ComEB, ComC and ComFA were specifically co-purified, and Maf was greatly enriched in the SPA-MreB eluate relative to the control strains (Table 1). Taken together, these results indicated that MreB is associated with several competence proteins in *B*. *subtilis*.

**Table 1:** MreB is in complex with competence proteins.

	NO SPA	PerR-SPA	SPA-MreB
ComGA	0,11	0,29	1,21
Maf	ND	0,25	1,00
ComEB	ND	ND	0,44
ComC	ND	ND	0,33
ComFA	ND	ND	0,32

Competence proteins eluted and quantified by LC-MS/MS in 3 independent SPA purification experiments using cells expressing SPA-MreB (NC66) or cells expressing no SPA-tagged protein (NC60) or PerR-SPA (NC135) as controls. Samples were taken at T2 (2 hours after the entry in stationary phase). Numbers in the table correspond to the Protein Abundance Index (PAI), established according to [40]. Values are normalized to the total amount of peptides detected in each experiment. ND stands for Not Detected.

**Fig 2:**
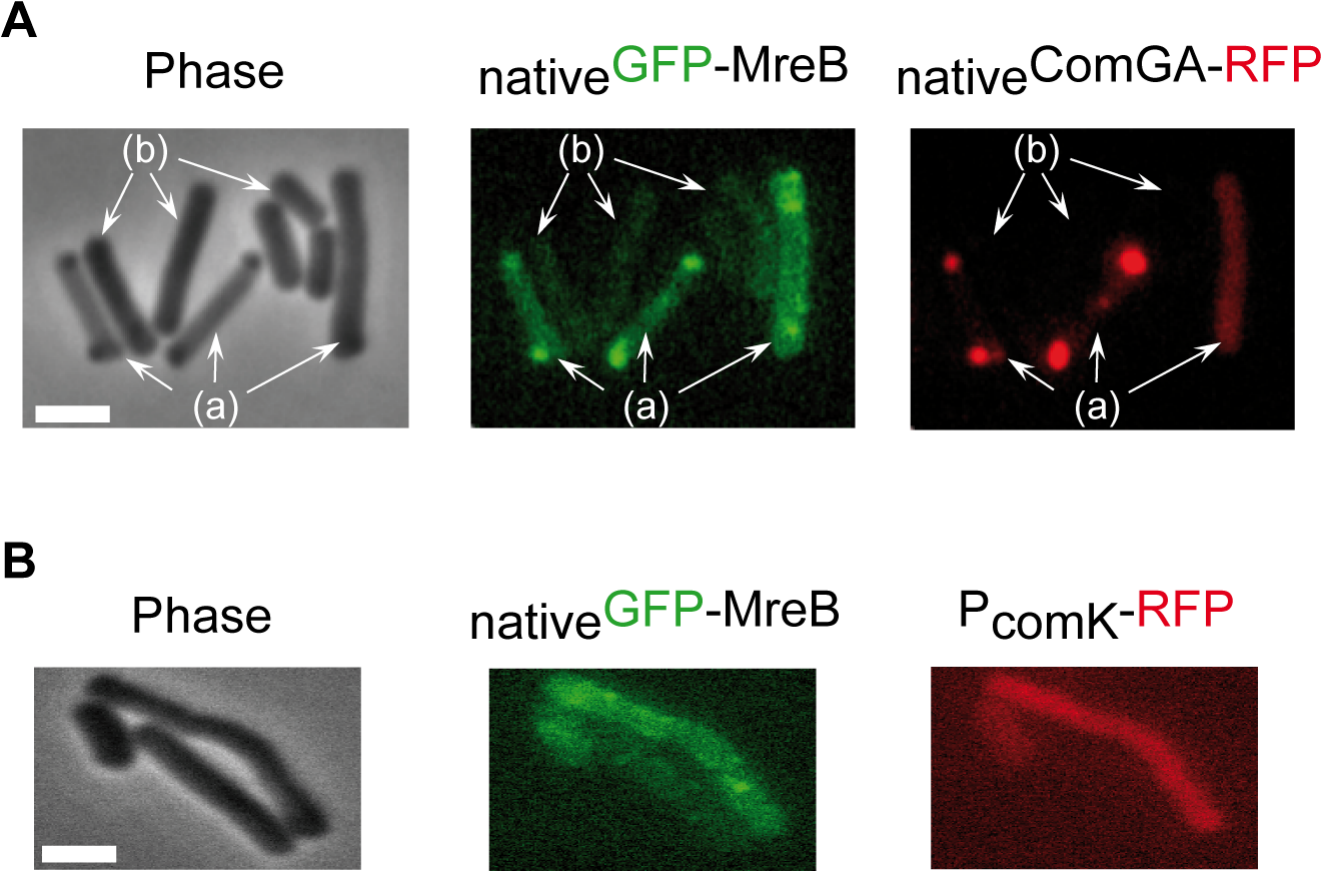
MreB co-localizes with ComGA polar clusters in competent cells. A. Co-localization of _native_GFP-MreB and _native_ComGA-RFP (strain NC121) in competent (displaying a RFP signal, a) and non-competent cells (b) of *B*. *subtilis*. Cells were grown at 37°C in competence medium to T_2_ and imaged by conventional epifluorescence microscopy. Typical group of cells imaged by (left to right): Phase contrast (Phase); GFP (green) and RFP (red) channels. The co-localization experiment of _native_Mbl-GFP and _native_ComGA-RFP is shown in S4D Fig as control. Scale bar, 1µm. B. Localization of _native_GFP-MreB in the *comGA* mutant background (strain NC215) at T_2_. The competent cells are easily identifiable as they express RFP under the control of the *comK* promoter. Scale bar, 1µm.

Next, we determined whether MreB displays a specific localization in the subpopulation of competent cells. We have shown that expression of *mreB* is complex, driven from three different promoters (Fig 1). To avoid a possible artifact of overexpression and/or misregulation, we replaced *mreB* by *gfp-mreB* at the native locus expressed under control of the native *mreB* regulatory sequences (P_native_ *gfp-mreB*), without leaving any scar or resistance cassette in the vicinity (see Methods for details, S4A Fig and S1 Movie). We then analyzed _native_GFP-MreB localization in cells that natively expressed a functional ComGA-RFP fusion (P_native_ *comGA-rfp*) as a marker for competence. Strikingly, in stationary phase cells (T_2_), most _native_GFP-MreB signal disappeared from the membrane and became diffuse in the cytoplasm (‘b’ cells in Figs 2A and S4C). In competence-expressing cells at T_2_, in addition of exhibiting a diffuse signal, _native_GFP-MreB formed clusters at one or both cell poles (‘a’ cells in Figs 2A and S4C). All polar MreB clusters (n > 200) were found to co-localize with ComGA polar clusters, while no _native_GFP-MreB signal was found in 15% (n > 200) of ComGA polar assemblies (Figs 2A and S4C). In the absence of *comGA*, MreB polar clusters were never observed and _native_GFP-MreB fluorescence signal was diffuse in all cells (n > 200) (Fig 2B). Control experiments showed that this co-localization was not due to bleed-through of the bright ComGA-RFP signal into the GFP channel (S4E Fig). In a given field of view, the integrated fluorescence signal of _native_GFP-MreB per cell was more than 3 times higher in competent (AU = 67.2 ±17.6, n = 102) than in non-competent (AU = 20.8 ±11.6, n = 103) cells, indicating that ComK-dependent expression of *mreB* (Fig 1) leads to increased levels of MreB protein in competent cells.

In exponential growth, a functional _native_Mbl-GFP fusion displayed the characteristic ‘motile patches’ localization (S4B Fig and S2 Movie). However, in contrast to _native_GFP-MreB, at T_2_ a _native_Mbl-GFP fusion was still localized in membrane-associated patches (albeit no longer motile, S4 Movie) along the sidewalls, which did not co-localize with ComGA polar clusters (S4D Fig). Thus, in competent cells MreB, but not Mbl, relocalizes into polar clusters that colocalize with, and are dependent on, the multi-functional competence protein ComGA.

### MreB is not required for genetic transformation

ComGA was first described for its essential role in natural genetic transformation [2]. We then tested if MreB could play a role during this process. Strikingly, transformation efficiency of in-frame *mreB* null mutants was increased about a hundred fold relative to the wild-type strain, while *mbl* and *mreBH* mutants had transformation efficiencies comparable to that of the wild-type (Table 2). However, in cells lacking *mreB*, both the percentage of competent cells and the timing of competence development were not affected (S5A and S6A Figs respectively).

**Table 2:** Transformation efficiencies.

Strain	Transformation efficiency	Standard deviation	Ratio
168, 5 mM	7,86E-06	2,98E-06	1,00
168, 25 mM	8,12E-06	2,91E-06	1,03
3725 (Δ*mreB*, Kan) 5 mM	9,03E-04	3,37E-04	114,91
4281 (Δ*mreB*, Cm) 5 mM	6,50E-04	2,89E-04	82,69
3725 (Δ*mreB*, Kan) 10 mM	5,55E-05	3,07E-05	7,06
3725 (Δ*mreB*, Kan) 25 mM	1,18E-05	3,37E-06	1,50
4261 (Δ*mbl*, Cm), 5 mM	6,91E-06	2,87E-06	0,88
2536 (Δ*mreBH*, Cm), 5 mM	4,24E-06	2,82E-06	0,54

The different *B*. *subtilis* strains studied were transformed using chromosomal DNA carrying a spectinomycin marker [35]. Each data point has been repeated at least 5 times which provides the standard deviation presented in the second column. The last column shows the ratio between each data point and the wild-type reference.

The final Mg^2+^ concentration present in the competence medium is given in mM.

High concentrations of magnesium (Mg^2+^) rescue the viability and shape defects of *mreBs* and other mutants involved in different aspects of cell wall synthesis by a yet unknown mechanism [14]. It has been proposed that Mg^2+^ may stiffen the cell wall, compensating for structural defects associated to the absence of *mreB* [41]. CM is traditionally supplemented with 5 mM Mg^2+^ [35]. Remarkably, increasing Mg^2+^ concentrations in CM progressively rescued the *mreB* transformation phenotype (Table 2). At 25 mM Mg^2+^, the transformation efficiency of *mreB* mutant cells was down to wild-type levels (Table 2). Taken together, these results suggested that the effect of MreB in transformation is indirect. They also raised the interesting possibility that specific cell wall defects could promote transformation in *B*. *subtilis*. One hypothesis was that the assembly or localization of the transformation apparatus across the cell wall was affected in the absence of *mreB*. To investigate this, we compared the localization of the transformation machinery in the wild-type and *mreB* mutant backgrounds using our _native_ComGA-RFP fusion. The dynamic localization of ComGA during competence has been extensively described [4]. In wild-type cells developing competence ComGA first appears diffuse in the cytoplasm (S7B Fig). Then, ComGA forms clusters associated to the inner face of the membrane with an important bias for the regions near the poles (S7C and S7D Figs), where it co-localizes with other main competence proteins to form the transformation machinery [4]. The number of ComGA focus per wild-type competent cell varies from one to nine, but the large majority of wild-type competent cells (41%, n>1500) display a single ComGA polar cluster (S5C and S5D Figs). The percentage of competent cells that displayed _native_ComGA-RFP clusters at T_2_ (S5B Fig) and among these the number of ComGA clusters (S5C Fig) were significantly higher in cells lacking *mreB* relative to wild-type cells. More specifically, the majority of *mreB* mutant competent cells (37%, n>1500) displayed three foci (S5C and S5E Figs). Expression of the *comGA* gene (S6B and S6D Figs) and ComGA protein levels (S6E Fig) were nevertheless unaffected in the *mreB* mutant. At high Mg^2+^ concentrations, mirroring the recovery of wild-type transformation efficiency of the *mreB* mutant, the distribution of the number of ComGA clusters per competent *mreB* mutant cell shifted back to wild-type levels (S5B Fig). We concluded that MreB is not directly required for natural transformation and that ComGA localization might be impacted by the cell wall integrity.

### ComGA inhibits cell elongation through MreB to prevent the escape from competence

**Fig 3:**
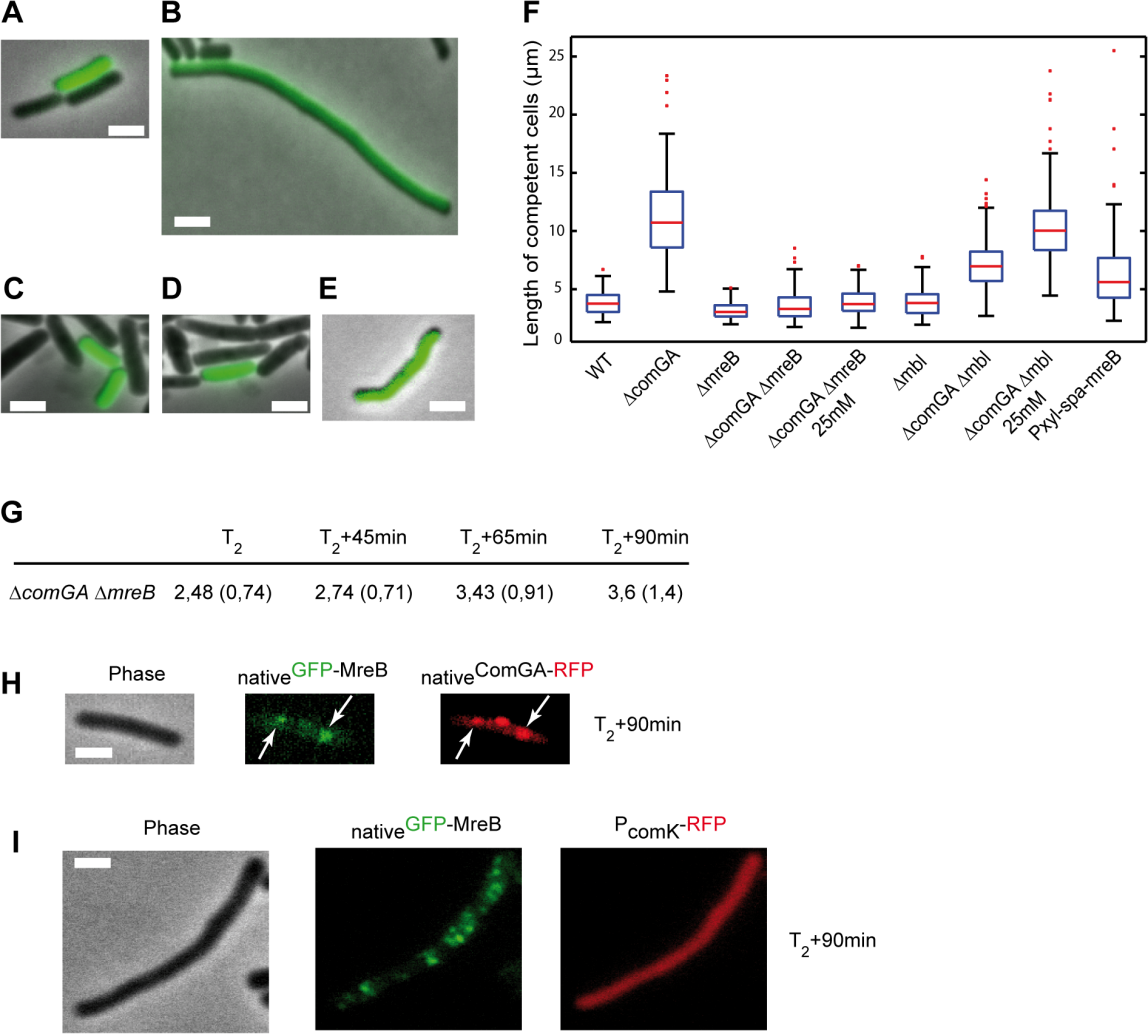
ComGA inhibits elongation during the escape from competence through MreB. A-E. Representative examples of the morphology of competent cells of the wild-type (NC59, A), the Δ*comGA* (NC164, B), the Δ*mreB* (NC161, C), the Δ*mreB* Δ*comGA* (NC169, D) and the P_xyl_-*spa-mreB* (NC197, E) strains 90 minutes after dilution into fresh competence medium (T_2_+90). Competent cells can be identified as they display _native_ComK-GFP signal. Scale bars, 2µm. F. Boxplots of the length of competent cells of the wild-type (NC59), Δ*comGA* (NC164), Δ*mreB* (NC161), Δ*mreB* Δ*comGA* (NC169), Δ*mbl* (NC162), Δ*mbl* Δ*comGA* (NC170) and Pxyl-*spa-mreB* (NC197) strains at T_2_+90 during the outgrowth experiment. Cells of the Δ*mreB* Δ*comGA* (NC169) and Δ*mbl* Δ*comGA* (NC170) strains were grown and diluted in normal competence medium (5 mM Mg^2+^) and in competence medium with a final Mg^2+^ concentration of 25 mM. At least 100 cells were counted for each strain and each condition. Details about this statistical analysis and the way this graph was constructed are presented in the Methods section. G. Mean cell length of competent cells of the Δ*mreB* Δ*comGA* strain (NC169) during the outgrowth experiment. Samples were taken for cell length measurements prior to dilution (T2) and 45, 65 and 90 minutes after dilution into fresh competence medium. At least 100 cells were measured for each time point. Standard deviations are indicated between brackets. H. Co-localization by epifluorescence microscopy of _native_GFP-MreB and _native_ComGA-RFP (NC121) after dilution and 90 minutes of growth (T_2_+90min). Left to right: Phase contrast (Phase); GFP (green) and RFP (red) channels. White arrows indicate the co-localizing MreB and ComGA clusters. Scale bar, 1 µm. I. Localization of _native_GFP-MreB in the absence of *comGA* during outgrowth. Cells expressing _native_GFP-MreB and P_comK_-RFP (as a marker of competence) in the *comGA* mutant background (strain NC215) were imaged by epifluorescence microscopy at T_2_+90min. Scale bar, 1µm.

It was shown that ComGA is also required for inhibition of cell elongation in cells exiting competence [11], while MreB directs cell elongation in exponentially growing cells [14,16,17]. We hypothesized that MreB could be involved in inhibition of cell elongation during competence escape through its association with ComGA. To test this, we performed outgrowth experiments using a ComK-GFP construct to distinguish competent from non-competent cells, as previously described [11]. Wild-type, Δ*comGA*, Δ*mreB* and Δ*mreB* Δ*comGA* mutant strains were grown to T_2_, when maximal competence is achieved, and diluted 20-fold into fresh medium. Samples were taken prior to dilution (T_2_) and 90 minutes after dilution (T_2_+90) for size and morphology characterization. At T_2_ competent and non-competent cells were indistinguishable in length for all strains (S3 Table) [12]. At T_2_+90, non-competent cells had resumed growth and division [11,12]. Competent cells of the wild-type strain (Fig 3A) were only slightly longer than at T_2_ (S3 Table), confirming the previously reported growth limitation imposed during the escape from competence [11]. Δ*mreB* competent cells also remained in a growth-limited state after 90 minutes of outgrowth and were significantly shorter than wild-type competent cells (Fig 3C and 3F and S3 Table) as previously reported for exponentially growing *mreB* mutant cells [41]. In contrast, Δ*comGA* competent cells were filamentous and often bent (Fig 3B and 3F and S3 Table), indicating that ComGA directly or indirectly inhibits cell elongation during the escape of competence [11]. However, when *mreB* was knocked out in the Δ*comGA* mutant strain, the filamentous phenotype of Δ*comGA* competent cells was rescued, and the average cell length of the Δ*mreB ΔcomGA* mutant was similar to that of wild-type competent cells at T_2_+90 (Fig 3D and 3F and S3 Table).

Wild-type rod shape is restored in *mreB* and *mbl* null mutants by addition of 25 mM Mg^2+^ to the growth medium, while addition of 2.5 mM Mg^2+^ is sufficient to restore wild-type growth rate of *mreB* mutants [41,42]. Consistently, Δ*mreB* mutant cells were viable and displayed wild-type growth and moderate cell shape defects in classic CM (i.e. 5mM Mg^2+^) (S8 Fig and S5 Movie). To exclude an indirect effect due to the inability of *mreB*-like mutants to elongate properly at low Mg^2+^ concentrations, we repeated the outgrowth experiments in CM containing 25 mM Mg^2+^. At T_2_+90, the length of Δ*comGA* Δ*mreB* competent cells was similar in conventional CM (5 mM Mg^2+^) and in CM with 25 mM Mg^2+^ (Fig 3F and S3 Table). In contrast, deletion of *mbl* ameliorated but did not rescue the Δ*comGA* filamentous phenotype, and in the presence of 25 mM Mg^2+^ Δ*comGA* Δ*mbl* competent cells filamented like Δ*comGA* competent cells (Fig 3F). Taken together, these results indicated that MreB plays a direct role in the growth limitation imposed during the escape from competence. However, it was still plausible that in the absence of MreB, competent *ΔcomGA* cells did filament but started dividing during the 90 minutes of outgrowth, as previously shown for Δ*maf ΔcomGA* mutant cells [12]. If this was true, the average length of Δ*mreB ΔcomGA* double mutant cells would first increase (during filamentation) and then decrease (upon initiation of cell division) between T_2_ and T_2_+90. Measurement of the length of competent cells at different times during the outgrowth experiment showed that Δ*mreB ΔcomGA* cells length slightly but progressively increased their average length from T_2_ to T_2_+90 (Fig 3G), excluding that they had filamented and then divided.

These findings suggested that ComGA-mediated inhibition of cell elongation during the escape from competence also involves MreB. One possibility is that ComGA directly or indirectly sequesters MreB in competent cells to delay the initiation of cell elongation upon outgrowth. According to this prediction, over-production of MreB could totally or partially bypass the ComGA checkpoint and thus promote elongation of competent cells during outgrowth. Consistently, when native levels of MreB were increased by expressing the functional *spa-mreB* fusion (S3 Fig) in the presence of the endogenous copy of *mreB*, competent cells filamented in a manner similar to *ΔcomGA* cells after 90 minutes of outgrowth. The mean length of cells overproducing SPA-MreB was almost twice the mean length of wild-type cells and cell length distribution was much broader, with cells exceeding 20 µm in length (Fig 3F).

### ComGA inhibits re-localization of MreB to the sidewalls during outgrowth

We found that at the time of maximum competence (T_2_) MreB forms polar clusters that co-localize with ComGA polar clusters and are dependent on the presence of ComGA (Fig 2). In addition, our findings suggest that MreB is involved, alongside ComGA, in the inhibition of cell elongation during outgrowth. We then verified if the localization of MreB and ComGA was still correlated during the escape from competence. After 90 minutes of outgrowth, MreB polar clusters were still present and co-localized with ComGA clusters in wild-type competent cells (Fig 3H). However, in the filamentous Δ*comGA* competent cells, MreB had already re-localized into motile patches along the sidewalls (Fig 3I). These results suggested a direct correlation between ComGA-dependent polar localization of MreB and the absence of elongation during the escape from competence.

### Expression of *comGA* in exponentially growing cells affects cell growth and morphology

**Fig 4:**
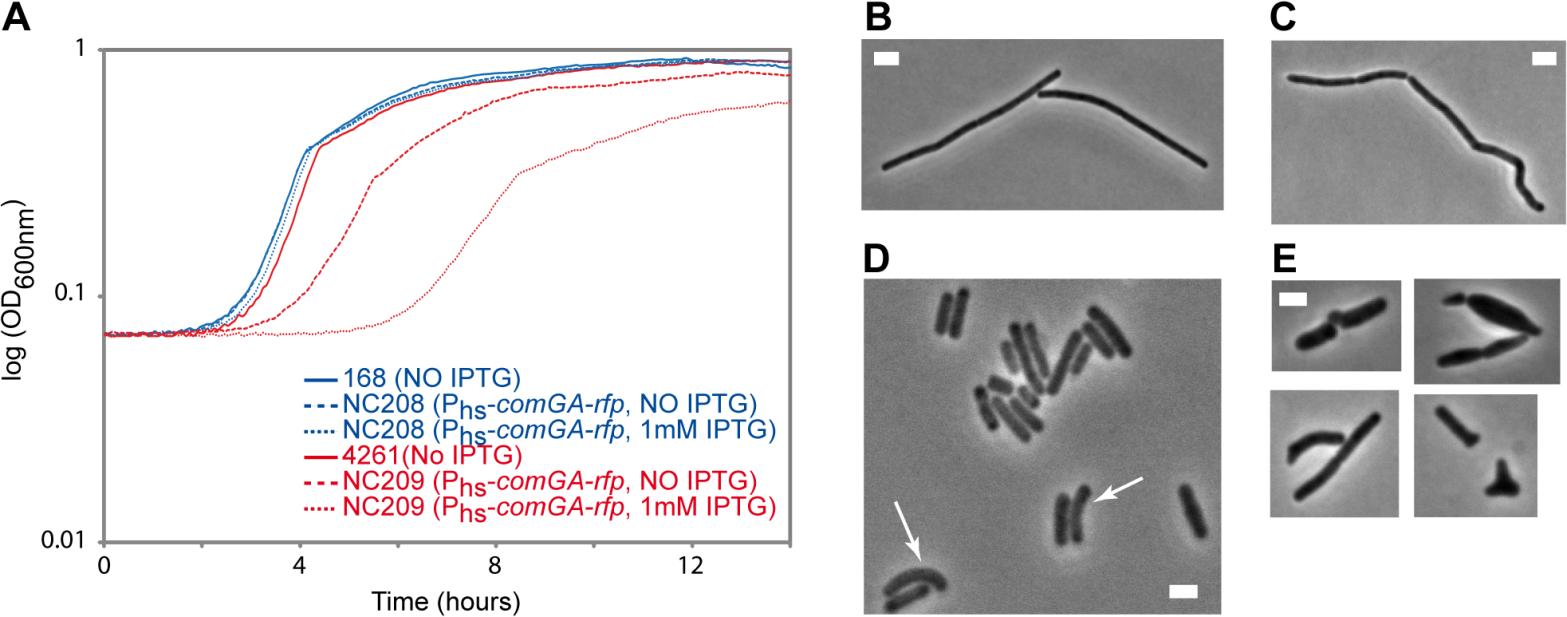
Effect of *comGA* expression during exponential phase. Growth and morphology of cells expressing *comGA* from the very strong (although poorly repressed) hyperspank promoter (*P*_*hs*_) in the wild-type and *mbl* mutant backgrounds. Cells were grown in LB medium at 37°C in the absence or in the presence of IPTG. A. Growth curves of the P_hs_-*comGA-rfp* (NC208, blue) and the *Δmbl* P_hs_-*comGA-rfp* (NC209, red) strains grown in the absence (NO IPTG, dashed lines) or in the presence (1mM IPTG, dotted lines) of inducer relative to the growth of their parental strains, 168 and 4261 (blue and red plain curves respectively). B-E. Phase contrast images of representative cells of the P_hs_-*comGA-rfp* (208, C) and *Δmbl* P_hs_-*comGA-rfp* (NC209, E) strains in the presence of 1 mM IPTG and their corresponding parental strains (B and D respectively) grown to OD_600_ = 0,15. Arrows in D point to *mbl* mutant cells that present morphological defects. Note that in D, the Δ*mbl* cells are in average shorter than wild-type cells (B). Scale bar, 2µm.

Our findings above suggest a model in which ComGA would directly or indirectly sequester MreB in competent cells. Unfortunately, difficulties to purify active recombinant MreB proteins currently unable biochemical work with MreB proteins of *B*. *subtilis* [14] and thus the direct interaction between MreB and ComGA could not be tested *in vitro*. No direct protein-protein interaction between MreB and ComGA was detected in pairwise yeast two-hybrid assays using full-length proteins (S9 Fig). False negatives are nevertheless frequent in two-hybrid assays [43], and thus the absence of interaction in yeast did not exclude a true protein interaction. Alternatively, we analyzed the effect of expression of *comGA* during exponential growth. In wild-type cells, *comGA* is exclusively expressed during competence [12]. In the same background, unnatural expression of *comGA* during exponential phase from an inducible promoter, was reported to have no effect on growth [11]. However, we reasoned that defects due to the sequestration of MreB by ComGA could be masked by the partial functional overlap between the three MreB isoforms [22]. Thus, we analyzed the effect of over-expression of *comGA* from the very strong (although poorly repressed) hyperspank promoter (*P*_*hs*_) in both wild-type and *mbl* mutant cells growing in rich (LB) medium. The *mbl* mutant strain grew almost like the wild-type strain in LB (Fig 4A), and a low percentage of cells (11%, n = 400 at OD = 0.15) displayed mild morphological defects (arrows in Fig 4D). Expression of *comGA* in the wild-type background had virtually no effect on growth (Fig 4A) [11] and morphology (Fig 4B and 4C). However, growth of the *mbl* mutant carrying the P_hs_-*comGA-rfp* construct, was significantly affected both in the absence and (to a bigger extent) in the presence of inducer (Fig 4A). This result clearly indicated that over-expression of *comGA* is toxic in the absence of Mbl. Furthermore, the majority (65%, n = 400 at OD = 0.15) of *comGA*-overexpressing *mbl* mutant cells showed progressive bulging and aberrant morphologies including Y-shaped cells and polar bulges characteristic of *mreB* (but not *mbl*) mutant cells [22,44] (Fig 4E), indicating impairment of cell morphogenesis and explaining the lethal effects on growth. We concluded that when *comGA* is expressed in exponentially growing cells, MreB cannot fully compensate for the absence of Mbl. These findings were consistent with the hypothesis that ComGA sequesters MreB to prevent cell elongation and limit growth. We could not test the effect of expression of *comGA* on the localization of MreB in the *mbl* mutant background because GFP fusions to MreB do not support growth in a *Δmbl ΔmreB* background.

## Discussion

### Expression and localization of MreB are regulated in stationary phase and during competence

In bacterial cells, like in their eukaryotic counterparts, proteins localize to specific locations, often in a dynamic manner, during growth. Spatiotemporal localization of proteins is critical for their function and orchestrates cellular processes. In exponentially growing *B*. *subtilis* cells, the *mreB* gene is highly expressed and MreB assembles into membrane-associated patches that move processively around the cell to control sidewall elongation [16–20]. Here, we show that when *B*. *subtilis* cells enter stationary phase in competence medium, expression of *mreB* drastically decreases and MreB delocalizes from the membrane exhibiting a largely diffuse localization in the cytoplasm. Such transcriptional regulation of *mreB* and the disassembly of MreB patches from the membrane may inhibit deposition of peptidoglycan along the sidewalls during stationary phase. Additionally, we show that expression of *mreB* is reactivated in cells that develop competence. In competent cells, MreB relocalizes in polar clusters together with the late competence protein ComGA. Co-localization of MreB and ComGA at the cell poles persists for at least 90 minutes of outgrowth into fresh media. MreB subsequently relocalizes as motile patches along the sidewalls to reinitiate elongation. Altogether, these findings underline the importance of dynamic regulation of gene expression and protein localization for bacteria to adapt to changing environmental conditions.

### Localization of the transformation apparatus is affected by the integrity of the cell wall

In cells lacking *mreB*, transformation efficiency was increased a hundredfold and the number of membrane-associated ComGA clusters was significantly higher than in wild-type cells. Both phenotypes were however rescued by high Mg^2+^ concentrations, suggesting that (i) MreB is not directly required for natural transformation in *B*. *subtilis*, and (ii) assembly of the transformation apparatus might be affected by structural features of the cell wall, as Mg^2+^ has been proposed to rigidify weakened cell-walls [41]. The transformation apparatus, which includes a type IV pilus-like structure that traverses the thick cell wall and is required for binding and importing the transforming DNA [45], preferentially localizes near the poles at the junction between the cylinder and the polar caps [4]. This region represents the interface between the sidewalls, which are intensively reshaped during growth, and the almost inert cell wall at the poles. Interestingly, this region is also chosen by phage SPP1 to bind and inject its DNA into the cytoplasm of *B*. *subtilis* [46]. During infection, SPP1 has to irreversibly bind to its receptor, YueB, encoded by a putative type VII secretion system gene cluster in *B*. *subtilis* [47,48]. YueB extends across the cell wall and also localizes at the junction between the cylinder and the polar caps [46]. Thus, this structurally differentiated region of the cell wall may contain positional information for the assembly of structures that need to cross the cell envelope. Initial assembly of the transformation apparatus pilus-like structure at these sites could then direct the localization of cytoplasmic competence-induced proteins such as ComGA at the inner leaflet of the cytoplasmic membrane. Specific defects in the structure or the organization of the cell wall of *mreB* mutant cells may favor the assembly of additional transformation apparatus at ectopic sites. Consistently, it has been shown that the absence of *mreB* induced the apparition of multiple sites containing polar material in *E*. *coli* cells [49,50]. Furthermore, inactivation of MreB in *Pseudomonas aeruginosa* led to the mislocalization of a normally polar type IV pilus [51].

### A model for sequestration of MreB by ComGA to prevent cell elongation and delay the escape from competence

We show here that in competent cells *mreB* is specifically transcribed from the same promoter than *maf* and that MreB protein levels are increased relative to non-competent cells. Competent *comGA* mutant cells filament upon dilution into fresh medium [11]. These long *comGA* mutant cells are unable to divide because Maf is still present and inhibits cell division [12]. When *mreB* was deleted in a *ΔcomGA* background, competent cells did not filament during the early stages of competence escape. When *mbl* was deleted, the average length of *ΔcomGA* cells exiting competence was also slightly reduced. High Mg^2+^ concentrations fully rescued *ΔcomGA* cells elongation in the absence of *mbl* but not in the absence of *mreB*. Taken together, these findings indicate that elongation of cells escaping competence primarily depends on MreB and cannot be rescued by the redundant action of Mbl and/or MreBH. Mbl could nevertheless play a mild secondary role in this process. Consistently, a low level of transcription of *mbl* was detected during stationary phase at T_2_, while expression of *mreBH* was completely switched off. When *mreB* was overexpressed in a wild-type background, cells escaping competence exhibited a filamentous phenotype, like *ΔcomGA* cells. We hypothesize that in this condition excess of MreB can bypass the ComGA-mediated inhibition of elongation and activate cell wall synthesis. Finally, confirming the implication of the two proteins in order to limit cell elongation, MreB was found (i) in the same complex than several competence proteins and (ii) co-localizing with ComGA polar clusters at T_2_ and throughout the 90 minutes following dilution into fresh medium.

**Fig 5:**
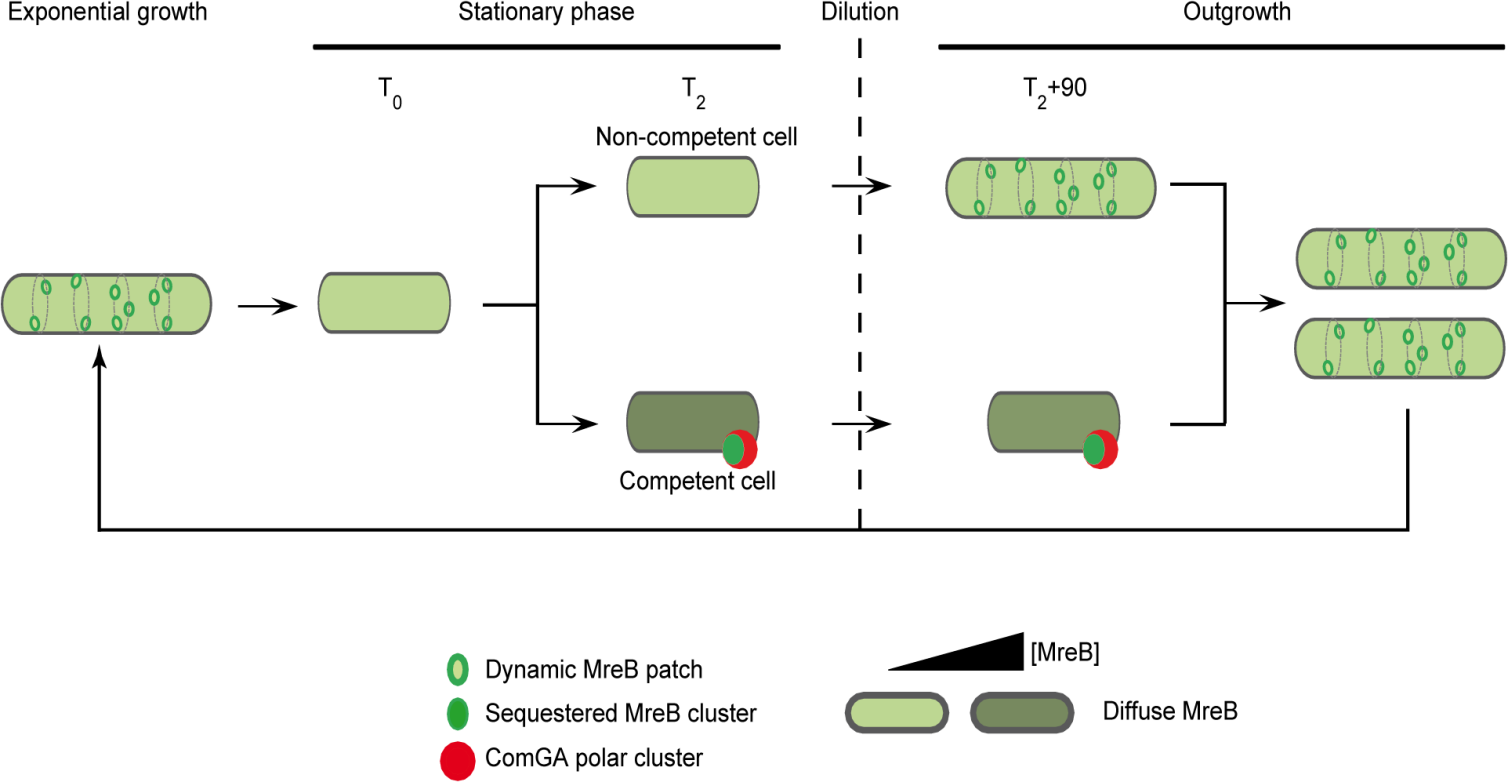
A model for sequestration of MreB by ComGA to prevent cell elongation and delay the escape from competence. In exponentially growing cells, MreB forms patches associated to the inner face of the lateral membrane in order to direct cell wall synthesis. As nutrient concentration decreases in the environment cells enter stationary phase (T_0_). Meanwhile, MreB proteins delocalize from the membrane to the cytoplasm of all the cells. Cell wall synthesis is therefore limited and the cells are in average shorter than during exponential growth. Throughout the two first hours following T_0_, competence develops in a low percentage of cells (2 to 10% in a wild-type background). When the maximum of competence is reached (T_2_), MreB concentration has increased in the competent cells. Moreover, in addition of being diffuse in the cytoplasm, MreB co-localizes with ComGA at the competent cells poles. When this stationary phase culture (T_2_) is diluted into fresh medium (outgrowth), the non-competent cells quickly start to elongate and divide as MreB re-associates with the sidewalls. At the opposite, competent cells growth is limited for more than 90 minutes as MreB is sequestered by ComGA at the poles. We finally hypothesize that as the ComGA lock is released (after 2 hours of outgrowth), MreB relocalizes at the membrane and allow cell elongation. We propose that a higher MreB concentration allows the competent cells to grow faster while exiting competence compensating for the delay previously imposed.

In the light of our results, we propose the model presented in Fig 5, in which ComGA inhibits cell elongation during the escape from competence by sequestering MreB, either directly or indirectly. No direct interaction between MreB and ComGA was detected in yeast two-hybrid assays and such interaction cannot be tested *in vitro* because active recombinant MreB of *B*. *subitlis* is currently not available for biochemical work [14]. However, we show here that expression of *comGA* in exponentially growing *mbl* mutant cells induces growth and morphological defects similar to those of *mreB* mutants. This result suggests that ComGA may be able to sequester MreB during exponential phase too. Therefore, if ComGA and MreB do not interact directly, then the potential protein(s) mediating their interaction during competence is (are) also expressed during vegetative growth. However, to date, all proteins found to co-localize with ComGA at the poles of competent cells are specifically over-produced during competence [4,52]. How the ComGA-MreB interaction is mediated remains an important question for future work.

### ComGA, a new regulator of the actin-like protein MreB?

General principles governing protein localization include capture by a cellular factor (e.g. interacting protein, DNA binding site, membrane domain or substrate) and self-assembly, where polymerization/depolymerization dictate the location of a protein at a given time [53]. Polymerization may also be regulated by binding proteins, like in the case of eukaryotic actin, where a myriad of actin-binding proteins (ABPs, [54,55]) regulate actin activity and dynamics. ABPs can nucleate, cross-link, bundle, anchor and regulate the state of polymerization of polymeric, filamentous actin (F-actin), and they can cap and stabilize the monomeric, globular actin (G-actin) pool in the cytoplasm.

Here, we show that in cells entering stationary phase, MreB dissociates from the sidewalls and becomes diffuse in the cytoplasm. Interestingly, it has been recently shown that the concentration of lipid-linked peptidoglycan precursors regulates the association of MreB to the membrane [56]. When precursors are depleted, MreB filaments disassemble into the cytoplasm. During the entry into stationary phase peptidoglycan precursor depletion probably occurs [57], as the metabolism slows down and the need of cell wall synthesis decreases, potentially explaining MreB relocalization. However, the details of the mechanism regulating the dynamic localization of MreB remain unknown. Numerous studies have identified a number of proteins that modulate FtsZ ring formation in *B*. *subtilis* [58–62] while the first ABP-like protein regulating MreB has yet to been found. It is plausible that one or several ABP-like protein(s), sensing the peptidoglycan precursor’s availability, promote MreB depolymerisation and/or stabilize the monomeric form of MreB in the cytoplasm.

In addition, we propose a model in which ComGA would sequester MreB in competent cells to prevent its localization to the sidewalls and therefore cell elongation. ComGA could then be considered as a new cellular regulator of the actin-like protein MreB. Only one protein that spatially regulates the MreB proteins has been reported in bacteria [63]. Indeed, the progressive depletion of RodZ leads to the misassembly of MreB into non-spiral structures before inducing a total loss of shape in *Escherichia coli* [63]. While RodZ can be considered as a positive regulator favoring the assembly of MreB at the right sites, ComGA could be classified as a negative regulator preventing the canonical MreB localization along cylindrical sidewalls.

### The specific increase of the amount of MreB in competent cells might compensate for the delay imposed by its sequestration

We suggest that sequestration by ComGA spatially regulates MreB during competence in *B*. *subtilis*. When ComGA is eventually degraded or inactivated allowing competent cells to resume growth [11], excess of MreB relative to non-competent cells would be free to rapidly form membrane-associated patches and initiate fast elongation. ComK-dependent induction of *mreB* expression during competence would therefore compensate for the timing disadvantage imposed by genetic transformation. The two levels of regulation (i.e. gene expression and protein localization) might generate and orchestrate the pathway controlling simultaneously a delay in growth and a way to compensate for it.

### One MreB isoform for one environmental adaptation?

It has been shown that MreB, Mbl and MreBH display partial functional redundancy in *B*. *subtilis* [22]. Overexpression of any one of the isoforms is sufficient to sustain lateral peptidoglycan synthesis and maintain cell shape in normal growth conditions. However, no single MreB isoform could support growth in various stress conditions, suggesting that multiplicity of MreB isoforms may become essential in specific environmental conditions [22]. Here we show that unlike *mreB*, *mbl* and *mreBH* are not specifically expressed during genetic competence. Consistently *mbl* and *mreBH* mutants displayed no competence-associated phenotypes. This specialization of MreB in competence further suggests that each isoform could be essential for specific environmental adaptations. A sigma-E sporulation specific promoter has been detected upstream *mbl* [26,30,36], while *mreBH* is part of the SigI regulon induced during heat stress [31,64]. Similarly to MreB in the context of competence, the localization and/or activity of Mbl and MreBH could be modulated by a regulator specifically produced during their respective adaptation. Future studies will reveal whether Mbl plays a role in sporulation and MreBH in stress response.

## Methods

### Microbiological methods

*Bacillus subtilis* strains were constructed by natural genetic transformation with selection for the appropriate antibiotic resistance marker. For transformation, competent cultures were prepared and incubated in competence medium (CM) with transforming DNA (~1 µg/ml) for 30 minutes at 37°C [35]. When needed, *B*. *subtilis* chromosomal DNA was prepared as detailed in [65]. Transformants were selected using 100 µg/ml spectinomycin, 10 µg/ml kanamycin, 5 µg/ml chloramphenicol, 16 µg/ml phleomycin and 1 µg/ml erythromycin. All the plates used to select transformants contained 25 mM of Mg^2+^. The details of all the new constructs in this publication are presented below. All new constructs were sequenced after introduction in the *B*. *subtilis* chromosome. *B*. *Subtilis* strains were grown in CM or LB media. When needed, the CM Mg^2+^ final concentration was increased to 25 mM. Strains are listed in S1 Table.

### Construction of promoter-luciferase fusion strains

Because some of our genes of interest are in the middle of operons, we decided to clone our constructs (promoter + RBS + luciferase) at the ectopic *amyE* locus.

Fragments of different lengths upstream the genes of interest (*mreB*, *mbl* and *mreBH*) and ending right before the genes RBS were amplified by PCR from the *B*. *subtilis* chromosome. To amplify the fragments P_*mreB123*_, P_*mreB23*_, P_*mreB3*_ (Fig 1A), P_*mbl12*_, P_*mbl2*_ (S2A Fig) and P_*mreBH1*_ (S2B Fig) we used the primers MCS-PmreB1-F and RBS-PmreB-R, MCS-PmreB2-F and RBS-PmreB-R, MCS-PmreB3-F and RBS-PmreB-R, MCS-Pmbl1-F and RBS-Pmbl-R, MCS-Pmbl2-F and RBS-Pmbl-R and MCS-PmreBH1-F and RBS-PmreBH-R respectively. In parallel, we amplified by PCR the upstream (amy-Front and choramphenicol cassette) and downstream (amy-Back and luciferase gene) *amyE* fragments from the plasmid pUC18cm-luc [66] using primers amyF-F and MCS-R and primers amyR-R and MCS-F respectively. Finally, using the Gibson method based on isothermal assembly [67], we joined the three fragments to obtain the PCR product “amy-F–Cm–Promoter–RBS–Luc- amy-R”. The final PCR product was used to transform strain NC57 by selection for cloramphenicol resistance.

Luciferase experiments were performed as we previously described in [68].

All primers are listed in S2 Table.

### Construction of the natively expressed *gfp-mreB* fusion

A method developed to construct scar-less and marker-less deletions in the genome of *B*. *subtilis* [69], was adapted to insert the *gfp* directly upstream *mreB*, at the native locus.

The first step was to delete, in the recipient strain (NC101, Neo^R^), the *radC* gene which is positioned right before *mreB*, by inserting a deletion cassette (Phleo^R^). The cassette was first amplified by PCR from plasmid pUC19-K7-010 [69] using the primers K7PH-F and K7PH-R. Then, the regions upstream (*radC* front) and downstream (*radC* back) the *radC* gene were amplified using the primers HindIII-Pmaf-F and Phleo-radC-R or Phleo-radC-F and HindIII-mreB-R, respectively. Finally, the three fragments were joined using the Gibson method [67] to obtain the following PCR product n°1:“*radC* front–Phleo cassette–*radC* back”. Transformation of the recipient strain (168 Δ*upp*) with this PCR product generated strain NC102 which is Neo^S^ and Phleo^R^.

Then, the deletion cassette was replaced by a fragment that re-introduced the *radC* gene and inserted *gfp* in front of *mreB*. This fragment was constituted by two blocks, namely P_*maf*_ *-maf-radC* (block 1) and *gfp*-*mreB* (block 2). These blocks were amplified by PCR using the following primers: Pmaf-F and GFP-radC-R (for block 1) and RBS-mreB-GFP-F and mreB-R (for block 2). The *gfp-mreB* block was amplified from chromosomal DNA of strain 3723 [41]. The two blocks were then joined using the Gibson method [67] to generate the PCR product n°2: P_*maf*_ *-maf-radC*-*gfp*-*mreB*. This final PCR product was used to transform the strain NC102 to obtain strain NC103 (Neo^R^ and Phleo^S^), which now contains *gfp* right in front of *mreB* inside its own operon. Cells of this strain (NC103) and its derivatives, in which Pnative-*gfp-mreB* is expressed as the only copy of *mreB* in the genome, were viable and displayed almost wild-type growth and morphology, indicating that the fusion is virtually functional (S4A Fig and S1 Movie).

All primers used are listed in S2 Table.

### Construction of the natively expressed *comGA-rfp* fusion

We decided to express the *comGA*-*rfp* fusion under the control of the native *comGA* promoter (P_*comGA*_) from the *thrC* locus. The Gibson method [67] was used to join four PCR fragments corresponding to the upstream (*thrC* front) and downstream (*thrC* back) regions of the *thrC* gene, the *comGA* promoter and orf, and the *mrfpruby* gene. These fragments were amplified using the primers hom-F and pDG1664-MCS-R (*thrC* front), pDG1664-MCS-R and thrB-R (*thrC* back), pDG1664-MCS-PcomGA-F and RFP-comGA-R (P_*comGA*_-*comGA*) and comGA-RFP-F and pDG1664-MCS-RFP-R (*mrfpruby*). The four fragments were joined to produce the final PCR product “*thrC* front–P_*comGA*_−*comGA*–*mrfpruby*- *thrC* back”. The thrC front and thrC back (which also contains an erythromycin resistance cassette) fragments were amplified from plasmid pDG1664 [70]. The *mrfpruby* gene was amplified from chromosomal DNA of strain RWSB5 [16]. The final PCR product was used to transform the NC57strain to generate strain NC118. In this strain and its derivatives, ComGA-mRFPruby displays the expected dynamic of localization during competence [4], indicating that the fusion is virtually functional (S5A–S5D Fig).

All primers are listed in S2 Table.

### Construction of the P_hyperspank_-*comGA-rfp* fusion

The method was comparable to the construction of the natively expressed *comGA-rfp* fusion described above. The Gibson method [67] was used to join four PCR fragments corresponding to the upstream (*thrC* front) and downstream (*thrC* back) regions of the *thrC* gene, the *comGA* gene and the *mrfpruby* gene. The P_*hyperspank*_ promoter was introduced through the *thrC* front fragment. The four fragments were amplified using the primers hom-F and pDG1664-MCS-R (*thrC* front), pDG1664-MCS-R and thrB-R (*thrC* back), pDG1664-MCS-comGA-F and RFP-comGA-R (*comGA*) and comGA-RFP-F and pDG1664-MCS-RFP-R (*mrfpruby*). The four fragments were joined to produce the final PCR product “*thrC* front–P_*hyperspank*_−*comGA*–*mrfpruby*- *thrC* back”. The thrC front (that contains the P_*hyperspank*_ promoter) and thrC back (that also contains an erythromycin resistance cassette) fragments were amplified from the pDP150 plasmid [71]. The *mrfpruby* gene was amplified from chromosomal DNA of strain RWSB5 [16]. The final PCR product was used to transform the wild type strain (168) to generating strain NC208.

All primers are listed in S2 Table.

### Construction of the P_comK_ *-rfp* fusion

We decided to clone the *rfp* gene under the control of the *comK* promoter at the *amyE* locus. The Gibson method [67] was used to join four fragments corresponding to the upstream (*amy* front) and downstream (*amy* back) regions of the *amyE* gene, the *comK* promoter and the *mrfpruby* gene. These fragments were amplified using the primers amy-F and PcomK-amy-R (amy front), RFP-amyR-F and amyR-R (amy back), amyF-PcomK-F and RFP-PcomK-R (PcomK) and PcomK-RFP-F and amyR-RFP-R (mrfpruby) respectively. The amy front and back (which also contains a spectinomycin resistance cassette) fragments were amplified from plasmid pDG1730 [70]. The *mrfpruby* gene was amplified from chromosomal DNA of strain RWSB5 [16]. The four fragments were joined to produce the PCR product “*amy* front–P_*comK*_−*mrfpruby*- *amy* back”. The final PCR product was used to transform the wild type strain (168), inserting the P_*comK*_- *mrfpruby* construct, at the *amyE* locus and selecting for chloramphenicol resistance.

All primers are listed in S2 Table.

### Construction of the P_xyl_-*spa-mreB* fusion

Translational fusion between the SPA-encoding (Sequential Peptide Affinity) and *mreB* open reading frame was cloned at the ectopic *amyE* locus under control of the xylose-inducible promoter P_xyl_ (pSG-SPA-Nter). pSG-Spa-Nter was generated by replacing the GFP contained in pSG1729 [72] by affinity purification tags (Sequential Peptide Affinity, or SPA) [39], right downstream from the P_xyl_ promoter. However to generate a N-ter fusion, the tags were inverted in comparison to the original SPA construct (i.e. Flag-TEV site-CBD). The inverted SPA tag was synthesized by Genscript. Then, the *mreB* open reading frame was PCR-amplified using primers ac-983/ac984, and cloned into the pSG-Spa-Nter vector, using the XhoI and EcoRI restriction sites. The resulting pAC637 plasmid (pSG-P_xyl_ *-spa*-*mreB*) was transformed into *B*. *subtilis* strain 4281 (Δ*mreB*::*cm*) and selected for resistance to spectinomycin, to obtain strain ABS1370. Finally, we used chromosomal DNA of strain ABS1370 to transfer by natural transformation the *amyE*::Pxyl-*spa*-*mreB* (Spc) construct in strain NC60 to obtain strain NC66.

### P_xyl_-*perR-spa* fusion

Chromosomal DNA from strain Bas013 [73] was used to transform the wild-type strain (168) and transfer the P_xyl_-*perR-spa* fusion. Chromosomal DNA of strain NC60 was then used to sequentially incorporate by natural transformation the mcComS and ComK-GFP constructs to generate the final strain NC135.

### Luciferase assay

Experiments were carried out as previously described [66,68]. In brief, the high instability of the luciferase, used as transcriptional reporter in *B*. *subtilis*, allows us to approach the measurement of a rate of expression [66], with a relatively small contribution from the cumulative effect of transcription. This particular characteristic of luciferase is in stark contrast with the behavior of other reporters, e.g. β-galactosidase. All the strains used in the luciferase experiments carried a multi-copy plasmid, mcComS [74], in order to increase the percentage of competent cells (from 2% to 35% in the wild-type background in the conditions used here, see Fig 4A).

For detection of luciferase activity, strains were first grown in LB medium to an optical density at 600 nm (OD_600nm_) of 2. Cells were then pelleted and resuspended in fresh competence medium, adjusting all the cultures to an OD_600nm_ of 2. These pre-cultures were then diluted 20 fold in fresh competence medium and 200 µl was distributed in each of two wells in a 96-well black plate (PerkinElmer). 10 µl of luciferin (PerkinElmer) was added to each well to reach a final concentration of 1.5 mg/ml (4.7 mM). The cultures were incubated at 37°C with agitation in a PerkinElmer Envision 2104 Multilabel Reader equipped with an enhanced sensitivity photomultiplier for luminometry. The temperature of the clear plastic lid was maintained at 38°C to avoid condensation. Relative Luminescence Units (RLU) and OD_600nm_ were measured at 2 minutes intervals. The data were plotted as RLU/OD (luminescence readings corrected for the OD) versus time from inoculation.

### Transformation efficiency measurements

*B*. *subtilis* strains were transformed using chromosomal DNA of strain BD4893 carrying a spectinomycin marker [35]. The number of transformants was evaluated by plating the transformed cultures on LB agar plates containing spectinomycin. Each transformation culture was also plated on non-selective LB agar in dilution series to establish the viable cell count. Transformation efficiency was calculated by dividing the number of transformants by the viable count of each strain.

### SPA-tag pull-down experiments

The strains containing the SPA fusions were grown to T_2_ in competence medium supplemented with 0.4% xylose (to induce the SPA fusions). The cultures were then centrifuged and promptly frozen in liquid nitrogen. The xylose concentration used was chosen in order to optimize the SPA fusions production and minimize the shape and growth phenotypes associated to the over-expression of MreB. The frozen cells pellets were then disrupted by cryogenic grinding (4 cycles of 2 minutes, always maintaining the cupules and the pellets in liquid nitrogen). The powder recovered from the grinding was resuspended in buffer A (Tris-HCl pH7,5 10 mM, NaCl 150 mM, EDTA 0,2 mM, Triton 0,1 mM and proteases inhibitors) and centrifuged to eliminate cell debris. SPA-MreB, PerR-SPA and No-SPA containing protein complexes were then isolated and analyzed as described in [75].

### Fluorescence microscopy

Cultures were grown in competence medium at 37°C from single freshly isolated colonies on plates containing the appropriate antibiotic selection. Samples for microscopic observation were taken at T_2_ (2 hours after the beginning of competence development) and T_2_+90 (90 minutes after dilution of a T2 culture in fresh competence medium) and immobilized on 1% agarose-coated microscope slides.

Bacteria were imaged with an inverted microscope (Nikon Ti-E) equipped with a 100× oil immersion objective and an environmental chamber maintained at 37°C. Conventional epifluorescence Images were recorded on phase-contrast and fluorescence channels (472/30-nm excitation filter and 520/35-nm emission filter for GFP, 562/40-nm excitation filter and 641/75-nm emission filter for RFP) with an ORCA-R2 camera (Hamamatsu). Images were processed with NIS-Elements (Nikon) software. Exposure time was set up to 200 ms for _native_GFP-MreB and 500 ms for _native_ComGA-RFP.

All TIRFM images were acquired on the same inverted microscope with a diode-pumped solid-state laser (Cobolt Calypso, 50mW, 491nm) and an Apo TIRF 100x oil objective (Nikon, NA 1.49). All images were collected with an electron-multiplying charge-coupled device (EMCCD) camera (iXON3 DU-897, Andor) with a gain of 300. Incidence angles and z-position were adjusted individually for all channels to obtain comparable evanescent wave penetration depth and focus position.

### Microfluidics

In order to follow *B*. *subtilis* growth over time, we used a microfluidic flow chamber technique (CellAsic part of EMD Millipore). The technology is divided in two parts: a perfusion control system and a microfluidic plate (specific for bacteria, B04A) that keeps cells in a single focal plan and allow us to induce and follow events during many generations.

The day before the experiment, strains were grown on selective plates. The next day, cells were resuspended in competence medium to OD = 1. 1µl of this resuspension was used to inoculate 1mL of fresh competence medium. Once the cultures reached early exponential phase, cells were injected in the chamber and incubated under a continuous flow (5µl/hour) of medium at 37°C.

### Quantification of the number of ComGA-RFP foci

In order to characterize ComGA-RFP foci at the single cell level, phase contrast and fluorescence images were taken simultaneously for cells grown to stationary phase (T_2_) in competence media. Fields of view of both images were used to generate sub-images displaying individual cells by applying a two-step algorithm. First, each single cell was detected by applying segmentation to phase-contrast images, resulting sub-images of individual cells with cell contours. Next, diffraction-limited comGA foci in each cell were identified in fluorescence images. Examples of individual cells are presented in S5 Fig Custom image processing codes (S10 Fig) were implemented in Matlab (Mathworks).

### GFP-MreB patches speed measurements

Kymograph analysis was applied to obtain the rotation speed of MreB patches as we previously described [16]. In brief, a series of parallel lines were created from one cell pole to the other (every other pixel), all perpendicular to the cell midline. Next, kymographs were generated, corresponding to movement of MreB patches at all positions along the cell longer axis. Finally, angles of the clear MreB traces on the kymographs were used to calculate the rotation speed.

### Competent cells length distribution

Length of competent cells was measured using the Metamorph software (Molecular Devices). Phase contrast images were used and the distance from one pole to the other was evaluated. Length of competent cells during the outgrowth experiment is shown as boxplots (refers to Fig 3F). The blue box edges indicate the first and third quartile while the red line indicates the median of the data set. In addition, the whiskers indicate the 5th and 95th percentiles and individual red points indicate outliers. All values with means, standard deviations (SD) and sample sizes are listed in S3 Table. Boxplots were plotted using Matlab 2013. The statistical significance of the differences observed is presented in S3 Table.

### Western blot

Whole cell extracts were fractionated by SDS-PAGE and transferred to a polyvinylidene difluoride membrane using a transfer apparatus according to the manufacturer’s protocol (Bio-Rad). After incubation with 5% nonfat milk in TBST (10 mM Tris, pH 8.0, 150 mM NaCl, 0.05% Tween 20) for 60 minutes, membranes were incubated with antibodies against GFP (1:10000) overnight at room temperature. Membranes were washed 3 times for 10 minutes with TBST and incubated with a 1:10000 dilution of anti-rabbit antibodies for 2h. Blots were washed with TBST three times and developed with the “ECL Prime” kit (Amersham) according to the manufacturer’s protocols. The Chemidoc system (Bio-Rad) was used to reveal the membrane and the Image Lab™ software (Bio-Rad) to analyze the intensity of the bands.

### Yeast two-hybrid assay

*Saccharomyces cerevisiae* cells expressing *B*. *subtilis* selected proteins as GAL4 BD fusions were mated with cells expressing either the same or another protein as GAL4 AD fusions as presented in [76]. For each fusion, two independent yeast clones were used. Binary interactions were revealed by growth of diploid cells after 5 days at 30°C on synthetic complete medium lacking leucine, uracil and histidine (to select for expression of the HIS3 interaction reporter, annotated-H). Specific interactions were reproduced independently at least three times.

## Supporting Information

S1 Fig Luciferase assay growth curves (referring to Fig 1). The growth curves corresponding to the luciferase assays presented in Fig 1 are shown: P_*mreB123*_ *-luc* (in red), P_*mreB23*_ *-luc* (in blue) or P_*mreB3*_ *-luc* (in green) in *comK*+ (A, strains NC91, NC92 and NC93 respectively) or *comK*- (B, strains NC146, 147 and 148 respectively) backgrounds. The black arrows denote the beginning of competence (T_0_).
(TIF)

S2 Fig Expression of *mbl* and *mreBH* are not competence-induced. A. Partial map of the *mbl* chromosomal vicinity. The two genes (*usd* and *spoIIID*) directly upstream *mbl* are represented as well as *mbl* itself. The two promoters identified in the region (P1 and P2) are represented by black flags. The two colored double-headed arrows delimit the fragments used in the luciferase assay to characterize the expression coming from each promoter. P_mbl12_ (in red) contains the two promoters while P_mbl2_ (in blue) only contains the last promoter in front of *mbl*, P2. B. Map of the *mreBH* operon. The only promoter present upstream *mreBH* (P1) is represented by a black flag. The red double-headed arrow delimits the P_mreBH1_ fragment (in red) used in the luciferase assay. C. Transcription profiles during growth in competence medium of strains expressing P_*mbl12*_ *-luc* (in red) or P_*mbl2*_ *-luc* (in blue) in a *comK*+ (solid lines, strains NC94 and NC95 respectively) or in a *comK*- (dotted lines, strains NC149 and NC150 respectively) background. The black arrows denote T_0_ and T_2_. D. Same as C except that the expression profiles were measured in a strain expressing P_*mreBH1*_ *-luc* (in red) in a *comK*+ (solid lines, strain NC96) or in a *comK*- (dotted lines, strain NC151) background. E. The growth curves corresponding to the luciferase assays presented in S2C Fig are shown: P_*mrbl12*_ *-luc* (in red) or P_*mbl2*_ *-luc* (in blue) in *comK*+ (NC94 and NC95 respectively) or *comK*- (NC149 in red and NC150 in blue respectively, dotted line) backgrounds. The black arrows denote the beginning of competence (T_0_). F. The growth curves corresponding to the luciferase assays presented in S2D Fig are shown: P_*mreBH1*_ (in red) in *comK*+ (NC96) or *comK*- (NC151, dotted line) backgrounds. The black arrows denote the beginning of competence (T_0_).
(TIF)

S3 Fig Functionality of the SPA-MreB fusion. Phase contrast micrographs of representative cells of the wild-type (168, A and B), the *mreB* mutant (3725, C and D) and the Δ*mreB*, P_xyl_-*spa-mreB* (ABS1370, E and F) strains grown to exponential (A, C and E) or stationary phase (B, D and F) at 37°C in competence medium. Cells of strain ABS1370 were grown in the presence of 0.4% of xylose to induce expression of SPA-MreB (E and F). Scale bars, 2 µm.
(TIF)

S4 Fig Subcellular localization of MreB and Mbl during exponential phase and competence. Localization of _native_GFP-MreB (strain NC121, A and C) and _native_Mbl-GFP (NC122, B and D) in exponentially growing cells (A and B) and in stationary phase cells (C and D). Cells were grown in competence medium at 37°C to T_2_. Thus, during stationary phase, some cells developed competence (and expressed the _native_ComGA-RFP fusion) and some didn’t (no RFP signal, see Fig 2A). The RFP fusion was imaged using conventional epifluorescence microscopy (EPI) while the GFP fusions were imaged using both EPI and TIRF microscopy (TIRFM). The corresponding Phase contrast (Phase) images of the EPI images are also shown. The TIRFM images are snapshots (200 ms exposure) of the movies presented as supplemental movies; S1 and S3 Movies for _native_GFP-MreB and S2 and S4 for _native_Mbl-GFP. Note that in panels C and D epifluorescence pictures were realized on competent cells while TIRFM S3 and S4 Movies were realized on non-competent cells. E. Control experiment showing that, under the image acquisition settings used in our experiments, there was no detectable bleed through between the RFP and GFP channels when imaging the _native_ComGA-RFP fusion. Strain NC118 was grown to T_2_ in competence medium and imaged by conventional epifluorescence microscopy. Phase contrast (Phase), RFP and GFP channels are presented. Scale bar, 2µm.
(TIF)

S5 Fig ComGA localization in wild-type and *mreB* mutant cells. A. Percentage of competent cells (displaying ComK-GFP signal) is shown in the wild-type (in red, NC60) and the *mreB* mutant (in blue, NC165) backgrounds. All samples were taken at T_2_. At least 4000 cells were counted for each condition. B. Percentage of ‘GA-localized’ cells (displaying at least one _native_ComGA-RFP focus) among the competent subpopulation is shown in the wild-type (in red, NC118) and *mreB* mutant (in blue, NC123) backgrounds. All samples were taken at T_2_. At least 1500 cells were counted for each condition. C. Histograms of number of _native_ComGA-RFP cluster per ‘GA-localized’ cell described as in B. Cells of the wild-type (in red, NC118) and the *mreB* mutant (NC123) strains were grown in conventional competence medium (5 mM final concentration of Mg^2+^) and in the case of the *mreB* mutant, in competence medium with a final concentration of Mg^2+^ of 25 mM. All samples were taken at T_2_. ComGA localization was characterized in at least 1500 competent cells for each strain in each condition. D and E. Examples of the main localization pattern of _native_ComGA-RFP at T_2_ in wild-type (C) and *mreB* mutant (D) cells growing in conventional competence medium (5 mM Mg^2+^). Epifluorescence images are converted to intensity map (a.u. stands for fluorescence intensity arbitrary unit) and the corresponding phase contrast images are presented. Examples are representative of the main population for each strain grown in 5 mM Mg^2+^ as shown in panel C (i.e. one polar cluster of ComGA in wild-type cells, three clusters for the *mreB* mutant strain). The white arrows point to ComGA-RFP clusters.
(TIF)

S6 Fig Competence regulation is not affected in the *mreB* mutant background. A–B. Transcription profiles during growth in competence medium of strains expressing *luc* from the promoters of *comK* (P_*comK*_ *-luc)* (A) and *comGA* (P_*comGA*_ *-luc*) (B). Expression of each fusion was measured in the wild-type (strains NC129 and NC175 respectively, in red) and *mreB* mutant (NC130 and NC176, in blue) backgrounds. Expression of the P_*comK*_ *-luc* construct in a *comK* mutant background (NC160, in green) is also shown in A as control. Black curves represent the growth (measured by OD_600_) of the wild-type strain during the experiment. The black arrows denote T_0_. C-D Growth curves of all the strains analyzed in the luciferase experiments shown in A (C) and B (D). E. Western blot showing the quantity of _native_ComGA-GFP in wild-type (wt, NC58) and *mreB* mutant (*ΔmreB*, NC203) cells grown to T_2_ in competence medium. Total protein extracts were blotted using anti-GFP antibody. The table shows the relative intensity of each band, calculated as the mean of 3 independent experiments (Standard deviation of the relative intensity is indicated between brackets). The wild type strain is used as a reference.
(TIF)

S7 Fig Localization of the _native_ComGA-RFP fusion. A-D. _native_ComGA-RFP imaged by conventional epifluorescence microscopy. Cells of strain NC118 were grown to T_2_ in competence medium at 37°C and imaged on agarose-coated slides. Representative phase contrast (left-hand panels) and corresponding heat map rendering micrographs (right-hand panels, a.u. stands for fluorescence intensity arbitrary unit) of a non-competent cell (A) and competent cells in which ComGA displays a diffuse localisation (B), one polar cluster (C) and multiple clusters (D). Note that because cells can induce competence during a relatively large window (1.5 to 2 hours, [68]), the different ComGA localizations (i.e. diffuse and localized at the poles) are all present at T_2_.
(TIF)

S8 Fig Growth curves of the wild-type (168, black) and the *mreB* mutant (3725, red) strains in conventional competence medium (5 mM Mg^2+^) at 37°C (refers to S5 Movie). (TIF)

S9 Fig Yeast two-hybrid assays between MreB and ComGA. Cells expressing in-frame fusions of the ORFs of *mreB* or *comGA* to the GAL4 binding domain (BD) fusions (left column) were mated with cells expressing fusions of *mreB* and *comGA* to the GAL4 activation domain (AD) (top line). For each strain two independent yeast clones were used to test interaction detection reproducibility. Binary interactions were revealed by growth of diploid cells on selective medium lacking histidine (-H). Negative controls (-) included BD and AD expressed from empty vectors. The MreB-MreB interaction was used as positive control.
(TIF)

S10 Fig Custom image processing codes (Matlab) used to quantify the number of ComGA-RFP foci per competent cell (presented in S5C Fig). (PDF)

S1 Table *B*. *subtilis* strains. (PDF)

S2 Table Primers used in this study. (PDF)

S3 Table Cell length during outgrowth. Table showing the mean length of competent and non-competent cells at T_2_ and the mean length of competent cells at T_2_+90 minutes. All the strains presented here carry a ComK-GFP fusion in order to differentiate the competent cells from the non-competent. Lengths are given in micrometers. The numbers in parentheses are standard deviations. No fewer than 100 competent cells were measured for each data point. ND stands for Not Determined. In the last column is presented a statistical test (Student’s paired t-test) revealing how significant is the size difference observed between competent cell of the wild-type and the other strains at T2+90. By conventional criteria, a two-tailed P value inferior to 0,0001 is considered to be extremely statistically significant. In contrast all the other P values are considered to be not quite statistically significant.
(PDF)

S1 Movie Visualization of _native_GFP-MreB patches dynamics (strain NC121) during exponential growth in competence medium at 37°C by TIRF microscopy. Exposure time was 200 ms and frame rate 1 image/sec over 2 minutes. _native_GFP-MreB patches rotate perpendicularly from the longitudinal cell axis with an average speed of 62.3nm/s (+/- 11). This movie refers to S3A Fig.
(AVI)

S2 Movie Visualization of _native_Mbl-GFP patches dynamics (NC122) during exponential growth in competence medium at 37°C by TIRF microscopy. Exposure time was 200ms and frame rate 1 image/sec over 2 minutes. This movie refers to S3B Fig.
(AVI)

S3 Movie Visualization of _native_GFP-MreB localization (NC121) in stationary phase in competence medium at 37°C by TIRF microscopy. Exposure time was 500 ms and frame rate 1 image/sec over 30 sec. This movie refers to S3C Fig.
(AVI)

S4 Movie Visualization of _native_Mbl-GFP localization (NC122) in stationary phase in competence medium at 37°C by TIRF microscopy. Exposure time was 500 ms and frame rate 1 image/sec over 30 sec. This movie refers to S3D Fig.
(AVI)

S5 Movie Bright field visualization of NC161 (*comK*::*comK*-*gfp*, *ΔmreB*) cells exponentially growing in conventional competence medium (5 mM Mg^2+^). Images were taken every 5 sec for 1 hour and 20 minutes. This time lapse experiment has been performed using microfluidics.
(AVI)

S6 Movie Bright field visualization of NC161 (*comK*::*comK*-*gfp*, *ΔmreB*) cells exponentially growing in competence medium with a Mg^2+^ final concentration of 25 mM. Images were taken every 5 sec for 1 hour. This time lapse experiment has been performed using microfluidics.
(AVI)
