# Supplementary figures and images for: MreB-Dependent Inhibition of Cell Elongation during the Escape from Competence in Bacillus subtilis
Source: PLoS Genet. 2015 Jun 19;11(6):e1005299. doi: 10.1371/journal.pgen.1005299 (PMC4474612; doi:10.1371/journal.pgen.1005299)

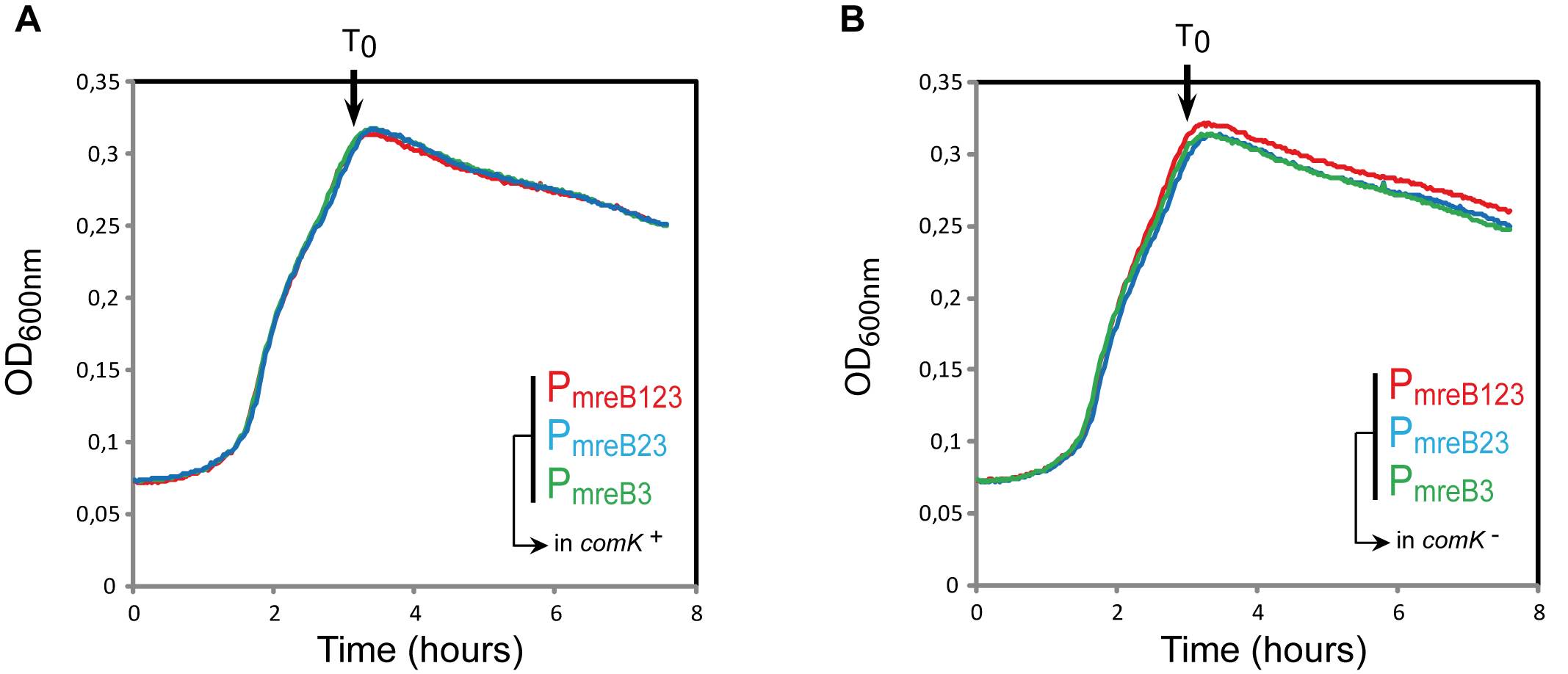

Supplement: S1 Fig — The growth curves corresponding to the luciferase assays presented in Fig 1 are shown: PmreB123 -luc (in red), PmreB23 -luc (in blue) or PmreB3 -luc (in green) in comK+ (A, strains NC91, NC92 and NC93 respectively) or comK- (B, strains NC146, 147 and 148 respectively) backgrounds. The black arrows denote the beginning of competence (T0). (TIF) [file pgen.1005299.s001.tif]

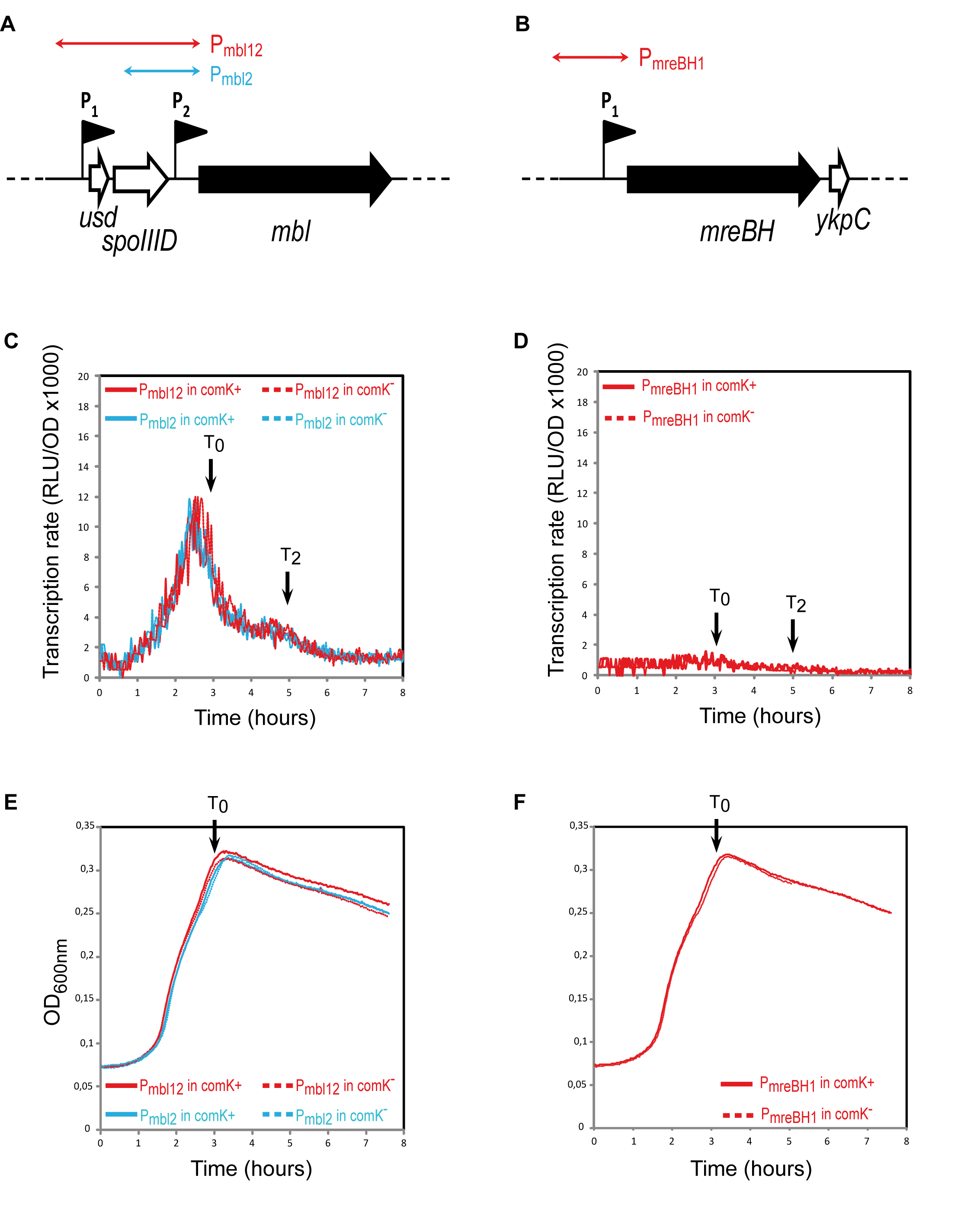

Supplement: S2 Fig — A. Partial map of the mbl chromosomal vicinity. The two genes (usd and spoIIID) directly upstream mbl are represented as well as mbl itself. The two promoters identified in the region (P1 and P2) are represented by black flags. The two colored double-headed arrows delimit the fragments used in the luciferase assay to characterize the expression coming from each promoter. Pmbl12 (in red) contains the two promoters while Pmbl2 (in blue) only contains the last promoter in front of mbl, P2. B. Map of the mreBH operon. The only promoter present upstream mreBH (P1) is represented by a black flag. The red double-headed arrow delimits the PmreBH1 fragment (in red) used in the luciferase assay. C. Transcription profiles during growth in competence medium of strains expressing Pmbl12 -luc (in red) or Pmbl2 -luc (in blue) in a comK+ (solid lines, strains NC94 and NC95 respectively) or in a comK- (dotted lines, strains NC149 and NC150 respectively) background. The black arrows denote T0 and T2. D. Same as C except that the expression profiles were measured in a strain expressing PmreBH1 -luc (in red) in a comK+ (solid lines, strain NC96) or in a comK- (dotted lines, strain NC151) background. E. The growth curves corresponding to the luciferase assays presented in S2C Fig are shown: Pmrbl12 -luc (in red) or Pmbl2 -luc (in blue) in comK+ (NC94 and NC95 respectively) or comK- (NC149 in red and NC150 in blue respectively, dotted line) backgrounds. The black arrows denote the beginning of competence (T0). F. The growth curves corresponding to the luciferase assays presented in S2D Fig are shown: PmreBH1 (in red) in comK+ (NC96) or comK- (NC151, dotted line) backgrounds. The black arrows denote the beginning of competence (T0). (TIF) [file pgen.1005299.s002.tif]

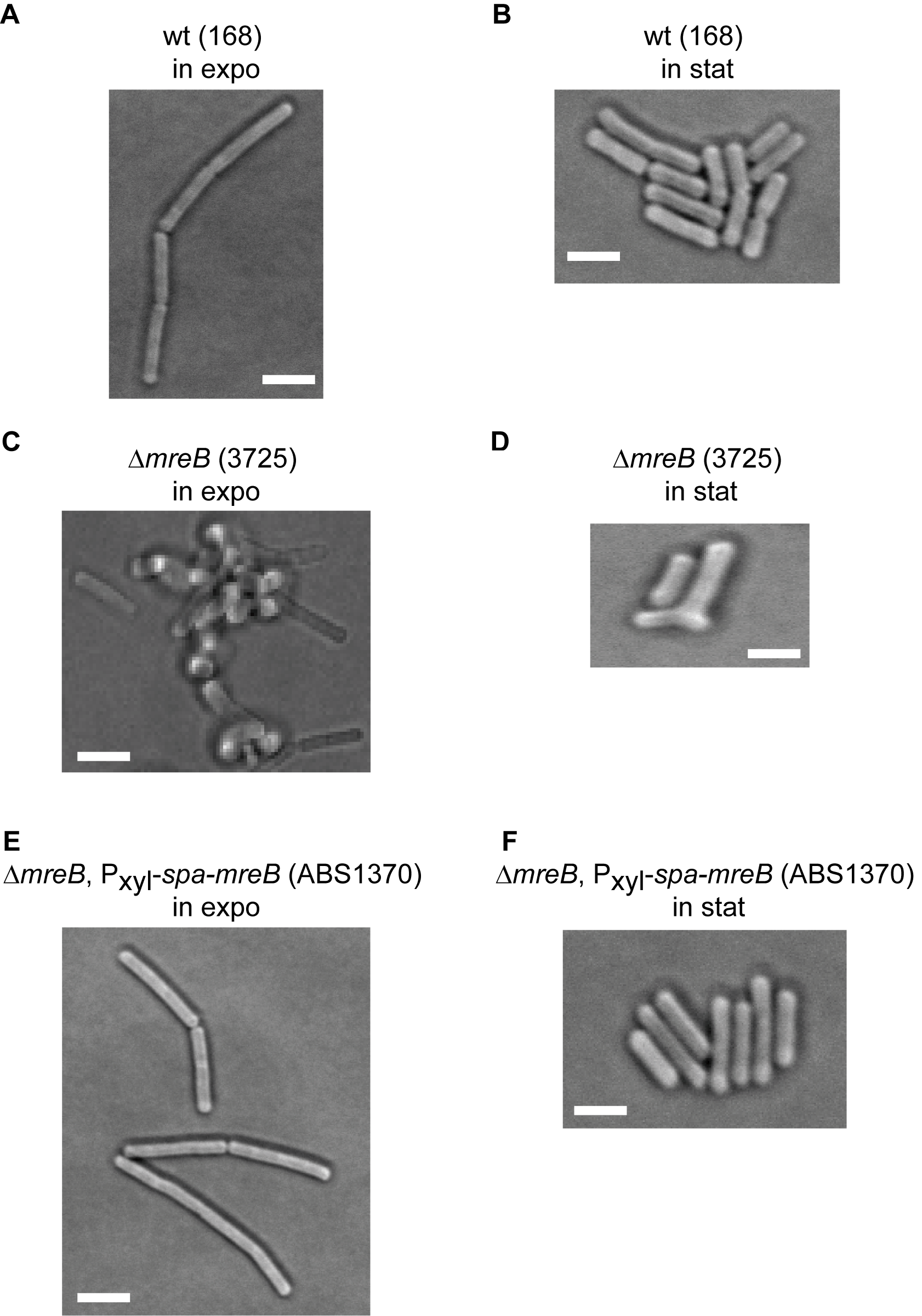

Supplement: S3 Fig — Phase contrast micrographs of representative cells of the wild-type (168, A and B), the mreB mutant (3725, C and D) and the ΔmreB, Pxyl-spa-mreB (ABS1370, E and F) strains grown to exponential (A, C and E) or stationary phase (B, D and F) at 37°C in competence medium. Cells of strain ABS1370 were grown in the presence of 0.4% of xylose to induce expression of SPA-MreB (E and F). Scale bars, 2 µm. (TIF) [file pgen.1005299.s003.tif]

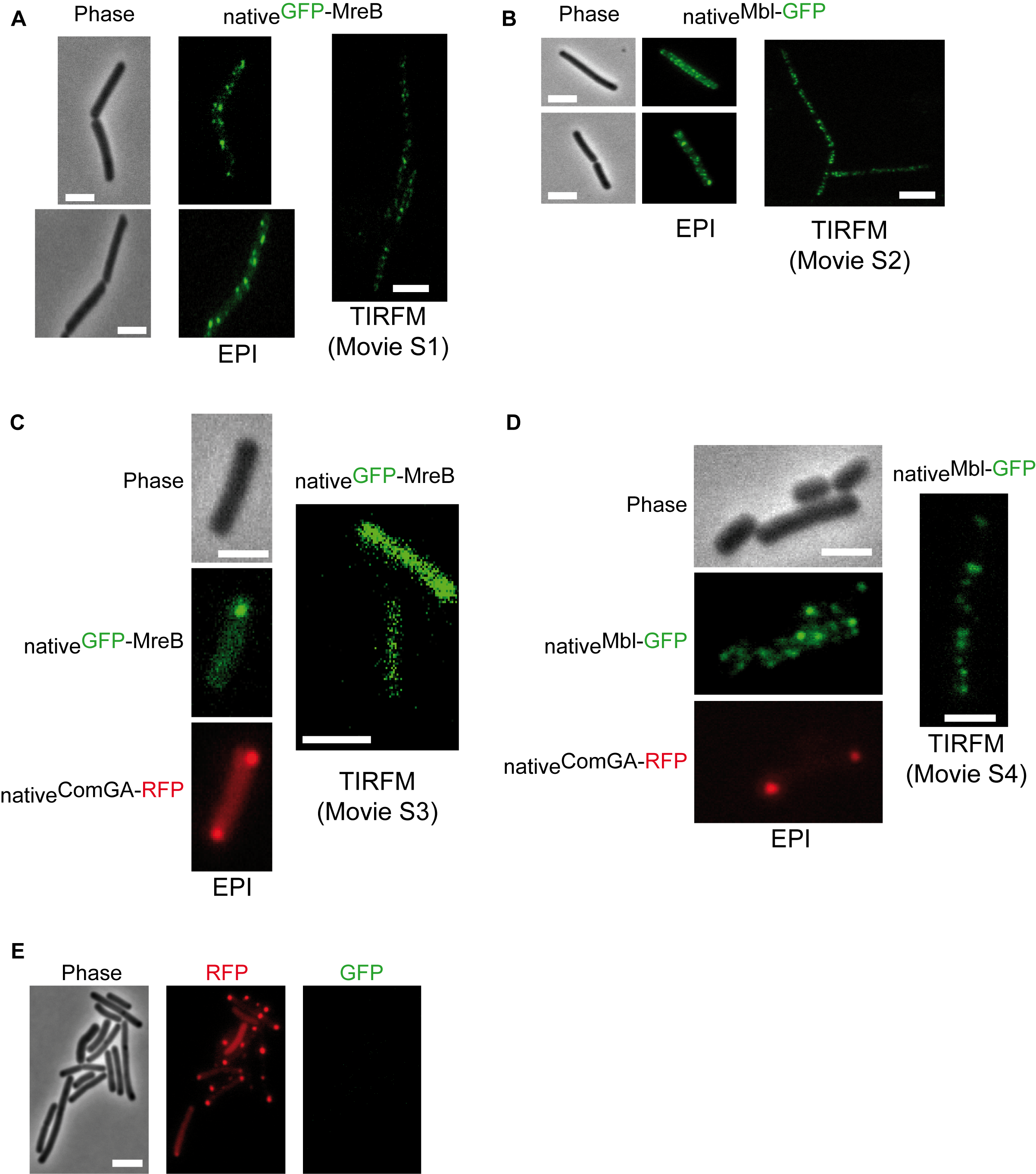

Supplement: S4 Fig — Localization of nativeGFP-MreB (strain NC121, A and C) and nativeMbl-GFP (NC122, B and D) in exponentially growing cells (A and B) and in stationary phase cells (C and D). Cells were grown in competence medium at 37°C to T2. Thus, during stationary phase, some cells developed competence (and expressed the nativeComGA-RFP fusion) and some didn’t (no RFP signal, see Fig 2A). The RFP fusion was imaged using conventional epifluorescence microscopy (EPI) while the GFP fusions were imaged using both EPI and TIRF microscopy (TIRFM). The corresponding Phase contrast (Phase) images of the EPI images are also shown. The TIRFM images are snapshots (200 ms exposure) of the movies presented as supplemental movies; S1 and S3 Movies for nativeGFP-MreB and S2 and S4 for nativeMbl-GFP. Note that in panels C and D epifluorescence pictures were realized on competent cells while TIRFM S3 and S4 Movies were realized on non-competent cells. E. Control experiment showing that, under the image acquisition settings used in our experiments, there was no detectable bleed through between the RFP and GFP channels when imaging the nativeComGA-RFP fusion. Strain NC118 was grown to T2 in competence medium and imaged by conventional epifluorescence microscopy. Phase contrast (Phase), RFP and GFP channels are presented. Scale bar, 2µm. (TIF) [file pgen.1005299.s004.tif]

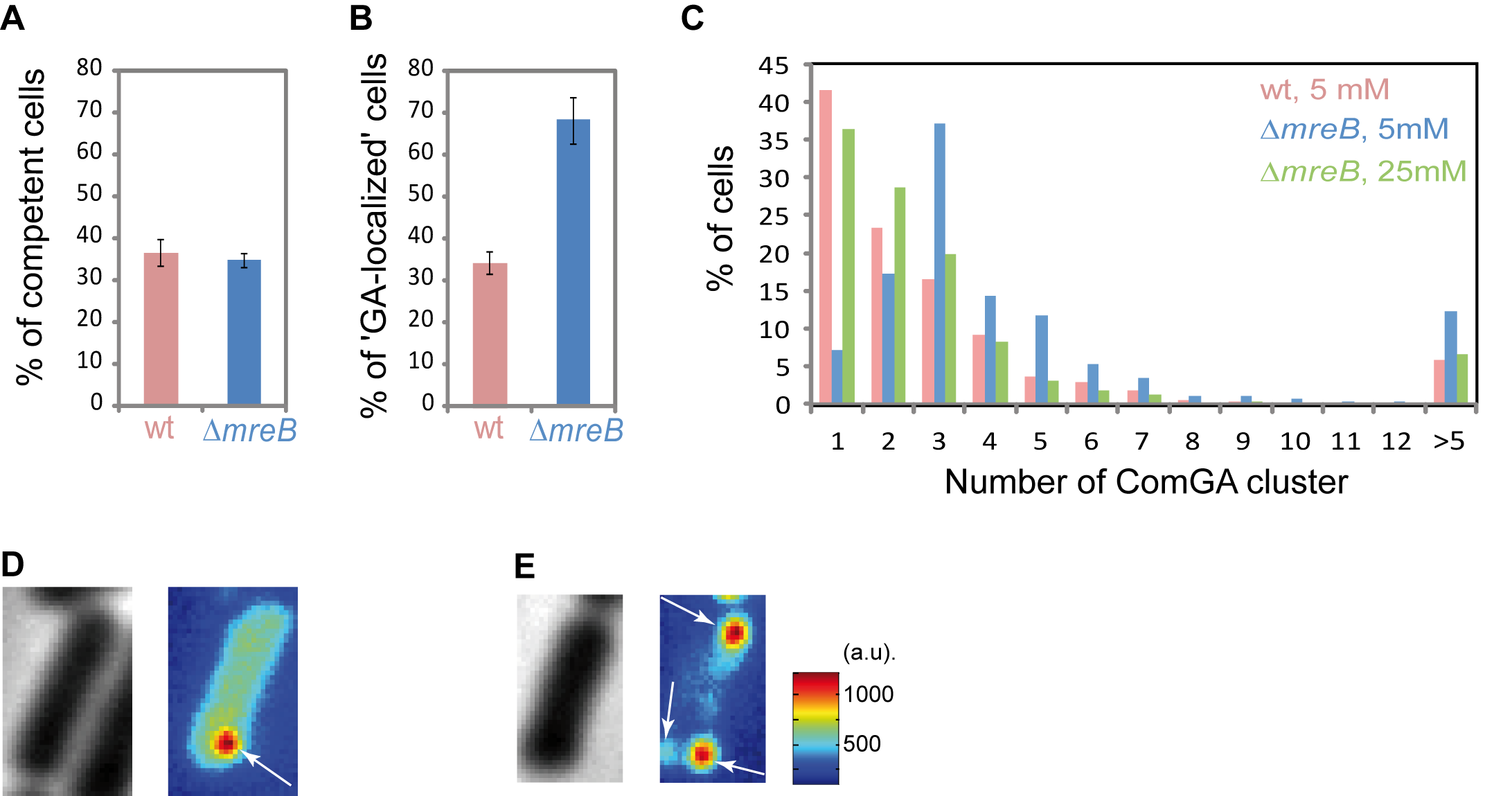

Supplement: S5 Fig — A. Percentage of competent cells (displaying ComK-GFP signal) is shown in the wild-type (in red, NC60) and the mreB mutant (in blue, NC165) backgrounds. All samples were taken at T2. At least 4000 cells were counted for each condition. B. Percentage of ‘GA-localized’ cells (displaying at least one nativeComGA-RFP focus) among the competent subpopulation is shown in the wild-type (in red, NC118) and mreB mutant (in blue, NC123) backgrounds. All samples were taken at T2. At least 1500 cells were counted for each condition. C. Histograms of number of nativeComGA-RFP cluster per ‘GA-localized’ cell described as in B. Cells of the wild-type (in red, NC118) and the mreB mutant (NC123) strains were grown in conventional competence medium (5 mM final concentration of Mg2+) and in the case of the mreB mutant, in competence medium with a final concentration of Mg2+ of 25 mM. All samples were taken at T2. ComGA localization was characterized in at least 1500 competent cells for each strain in each condition. D and E. Examples of the main localization pattern of nativeComGA-RFP at T2 in wild-type (C) and mreB mutant (D) cells growing in conventional competence medium (5 mM Mg2+). Epifluorescence images are converted to intensity map (a.u. stands for fluorescence intensity arbitrary unit) and the corresponding phase contrast images are presented. Examples are representative of the main population for each strain grown in 5 mM Mg2+ as shown in panel C (i.e. one polar cluster of ComGA in wild-type cells, three clusters for the mreB mutant strain). The white arrows point to ComGA-RFP clusters. (TIF) [file pgen.1005299.s005.tif]

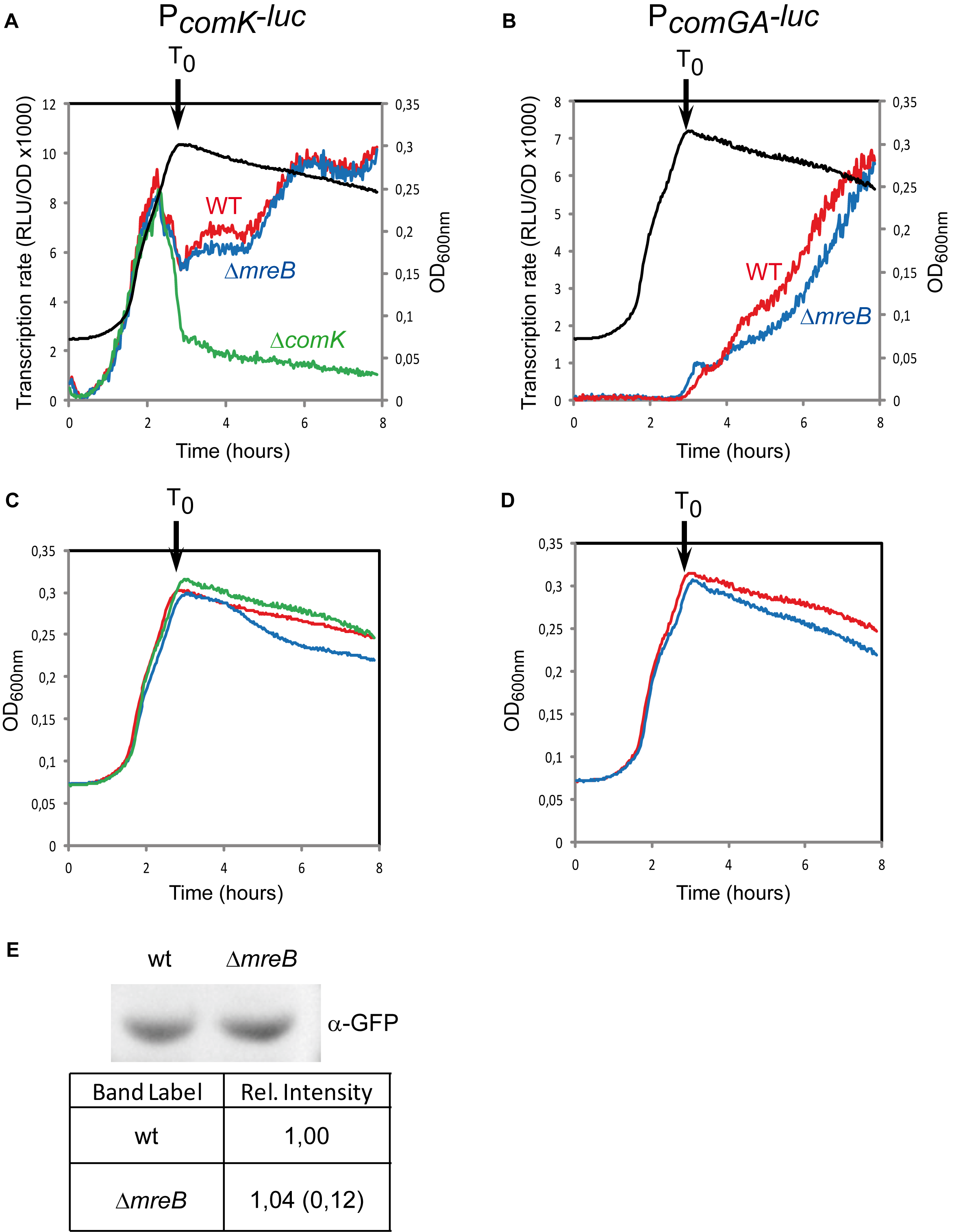

Supplement: S6 Fig — A–B. Transcription profiles during growth in competence medium of strains expressing luc from the promoters of comK (PcomK -luc) (A) and comGA (PcomGA -luc) (B). Expression of each fusion was measured in the wild-type (strains NC129 and NC175 respectively, in red) and mreB mutant (NC130 and NC176, in blue) backgrounds. Expression of the PcomK -luc construct in a comK mutant background (NC160, in green) is also shown in A as control. Black curves represent the growth (measured by OD600) of the wild-type strain during the experiment. The black arrows denote T0. C-D Growth curves of all the strains analyzed in the luciferase experiments shown in A (C) and B (D). E. Western blot showing the quantity of nativeComGA-GFP in wild-type (wt, NC58) and mreB mutant (ΔmreB, NC203) cells grown to T2 in competence medium. Total protein extracts were blotted using anti-GFP antibody. The table shows the relative intensity of each band, calculated as the mean of 3 independent experiments (Standard deviation of the relative intensity is indicated between brackets). The wild type strain is used as a reference. (TIF) [file pgen.1005299.s006.tif]

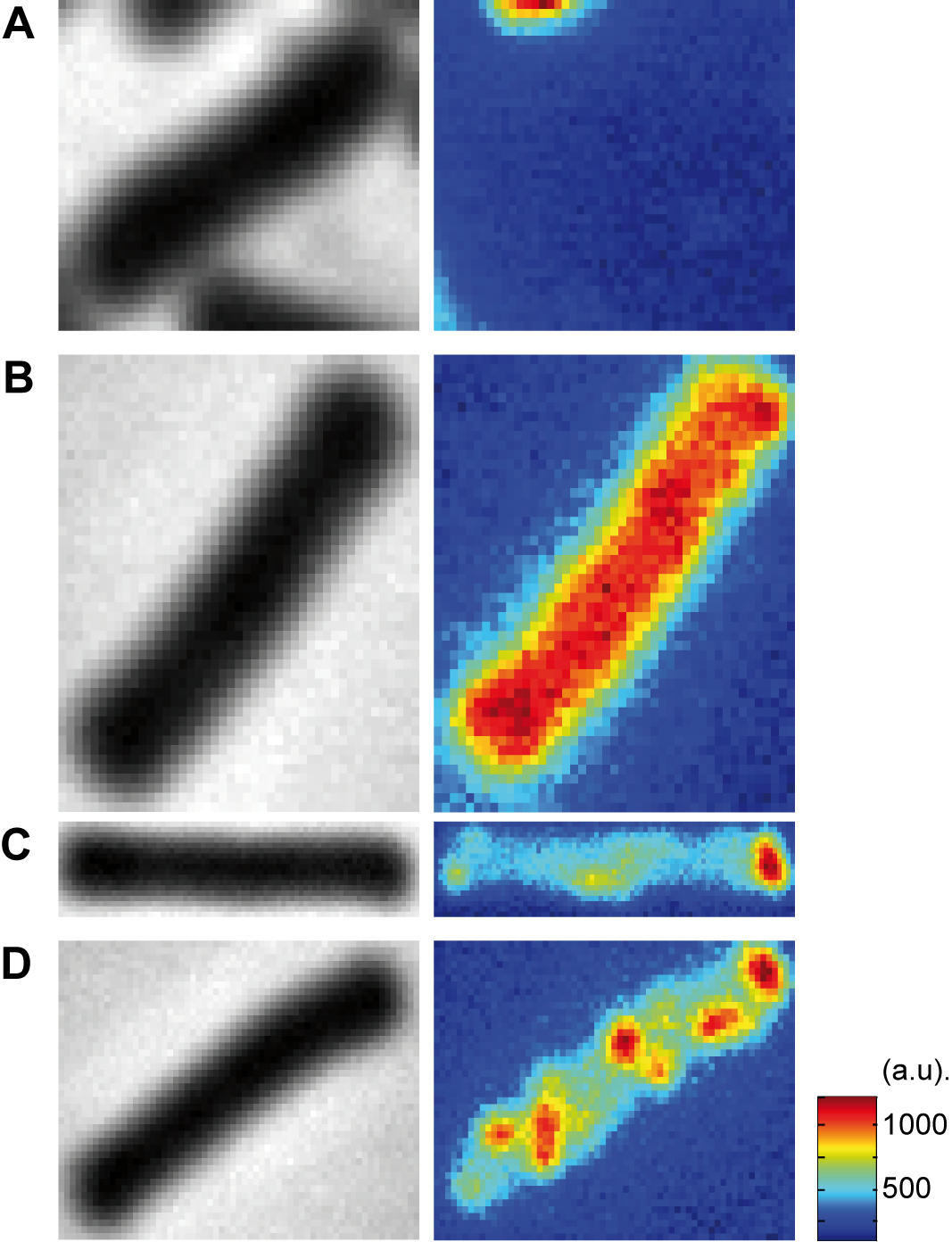

Supplement: S7 Fig — A-D. nativeComGA-RFP imaged by conventional epifluorescence microscopy. Cells of strain NC118 were grown to T2 in competence medium at 37°C and imaged on agarose-coated slides. Representative phase contrast (left-hand panels) and corresponding heat map rendering micrographs (right-hand panels, a.u. stands for fluorescence intensity arbitrary unit) of a non-competent cell (A) and competent cells in which ComGA displays a diffuse localisation (B), one polar cluster (C) and multiple clusters (D). Note that because cells can induce competence during a relatively large window (1.5 to 2 hours, [68]), the different ComGA localizations (i.e. diffuse and localized at the poles) are all present at T2. (TIF) [file pgen.1005299.s007.tif]

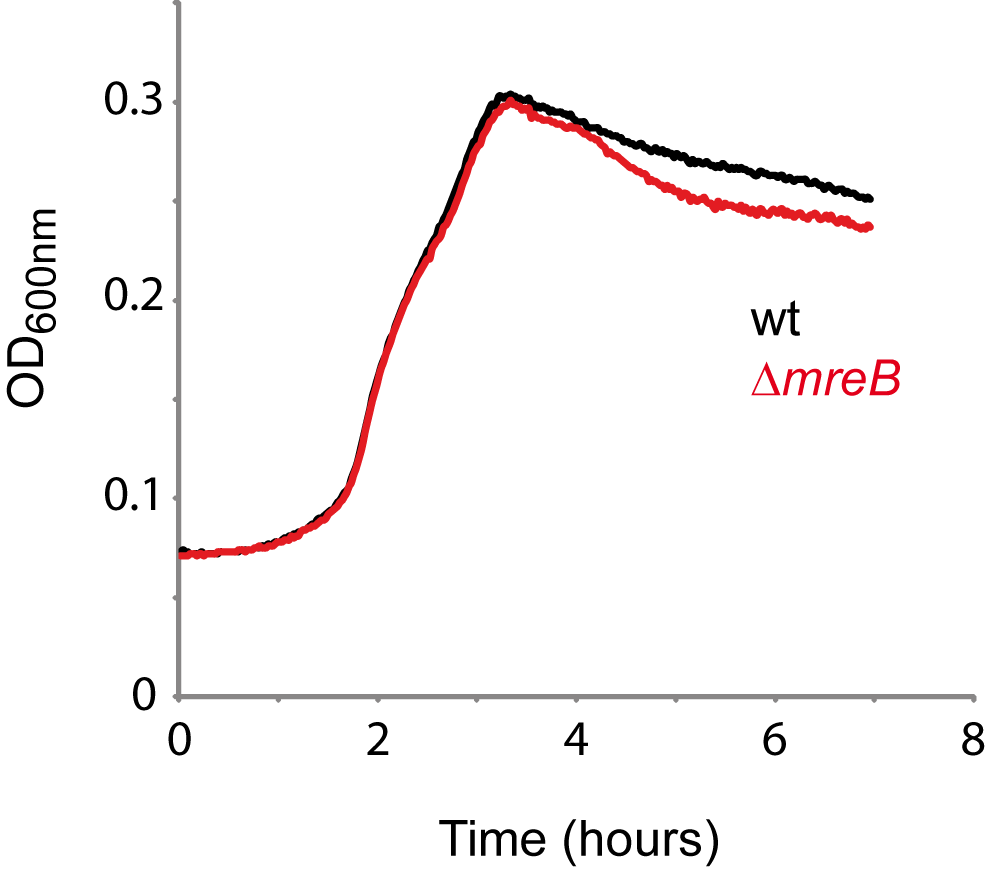

Supplement: S8 Fig — (TIF) [file pgen.1005299.s008.tif]

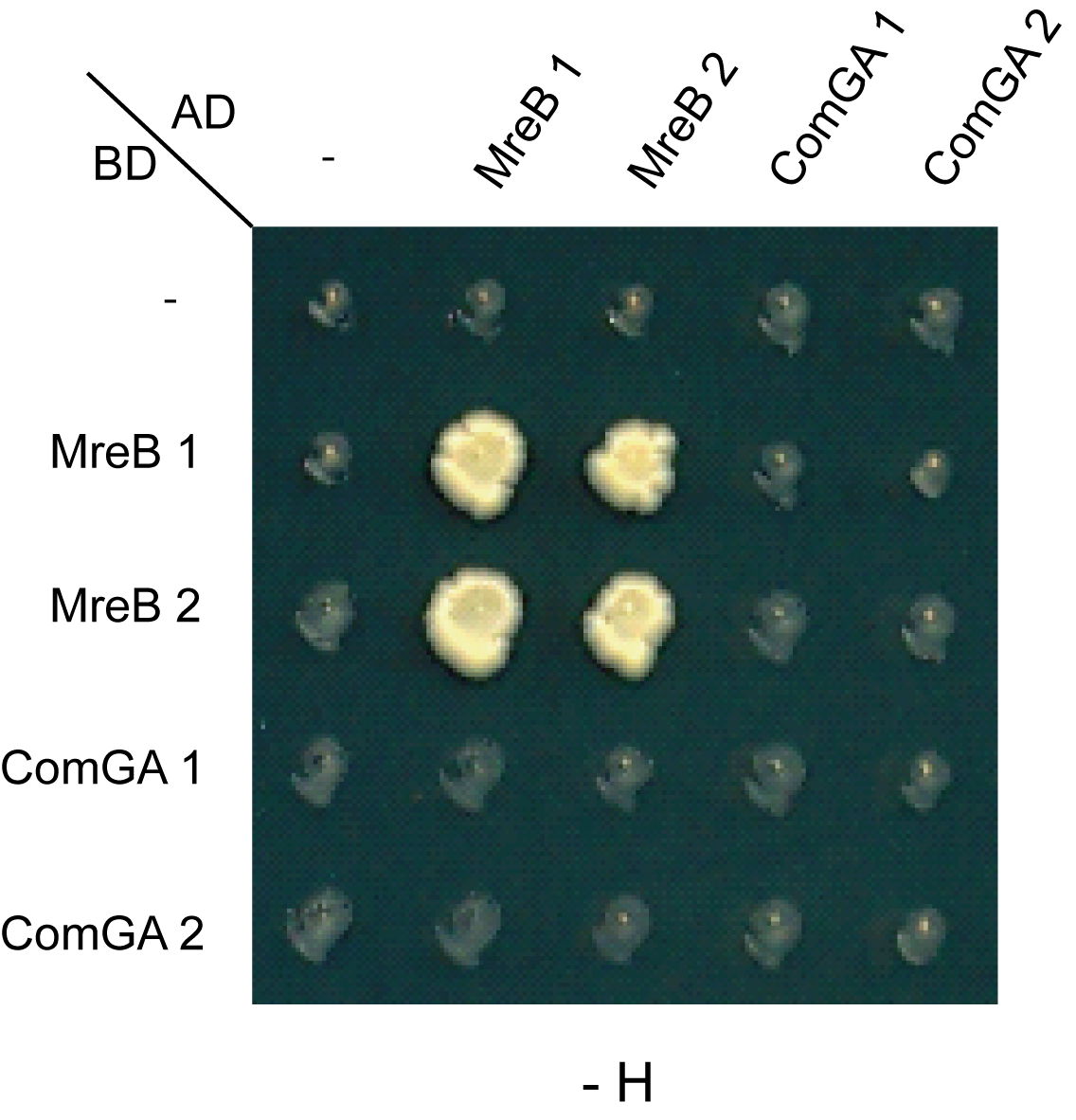

Supplement: S9 Fig — Cells expressing in-frame fusions of the ORFs of mreB or comGA to the GAL4 binding domain (BD) fusions (left column) were mated with cells expressing fusions of mreB and comGA to the GAL4 activation domain (AD) (top line). For each strain two independent yeast clones were used to test interaction detection reproducibility. Binary interactions were revealed by growth of diploid cells on selective medium lacking histidine (-H). Negative controls (-) included BD and AD expressed from empty vectors. The MreB-MreB interaction was used as positive control. (TIF) [file pgen.1005299.s009.tif]
