# Supplementary material for: MreB-Dependent Inhibition of Cell Elongation during the Escape from Competence in Bacillus subtilis
Source: PLoS Genet. 2015 Jun 19;11(6):e1005299. doi: 10.1371/journal.pgen.1005299 (PMC4474612; doi:10.1371/journal.pgen.1005299)
Supplement: S10 Fig — (PDF) [file pgen.1005299.s010.pdf]

**S10 Fig. Custom image processing codes (Matlab).**

'Quantification of the number of ComGA-RFP foci' (refers to S5 Fig.).

```
function [foci, cell]=measure_foci(I_phase, I_gfp)

I=I_phase;
I=max(max(I))-I;
% imshow(I,[])
background = imopen(I,strel('disk',5));
I2 = I - background;
I3 = imadjust(I2);

level = graythresh(I3);
bw = im2bw(I3,level*1.2);
bw = bwareaopen(bw, 100);

cc = bwconncomp(bw,8)
cc.NumObjects

labeled = labelmatrix(cc);
RGB_label = label2rgb(labeled, @spring, 'c', 'shuffle');
% figure, imshow(RGB_label)

L = bwlabel(bw,8);
I_phase_invert=max(max(I_phase))-I_phase;

for i=1: max(max(L))
    [r, c] = find(L==i);
    I_crop_phase=imcrop(I_phase_invert, [min(c)-5 min(r)-5 max(c)-min(c)+10
max(r)-min(r)+10]);
    I_crop_gfp=imcrop(I_gfp, [min(c)-5 min(r)-5 max(c)-min(c)+10 max(r)-
min(r)+10]);

    subplot(1,2,1), imshow(I_crop_phase,[],'Border','tight')
    title(sprintf('Number of cell: %g out of %g\', i,cc.NumObjects))
    subplot(1,2,2), imshow(I_crop_gfp,[],'Border','tight');
    hold on
    contour (I_crop_phase,[1000 1000],'g-','linewidth',3);
    colormap jet;

    for j=1:10
        [xi,yi,P] = impixel;
        plot(xi,yi,'g.','MarkerSize',30);
        if xi==1 || yi==1 || xi==2 || yi==2, break, end
        cell(i).foci(j)=P(1);
    end

    hold off
    pause
end

foci = [cell.foci]';

end
```
