# Supplementary material for: MreB-Dependent Inhibition of Cell Elongation during the Escape from Competence in Bacillus subtilis
Source: PLoS Genet. 2015 Jun 19;11(6):e1005299. doi: 10.1371/journal.pgen.1005299 (PMC4474612; doi:10.1371/journal.pgen.1005299)
Supplement: S1 Table — (PDF) [file pgen.1005299.s011.pdf]

S1 Table. *B. subtilis* strains.

Strain	Genotype	Source (1)
168	trpC2	(1)
3725	trpC2 Δ*mreB* (Kan)	(2)
4281	trpC2 Δ*mreB* (Cm)	(3)
2536	trpC2 Δ*mreBH* (Cm)	(4)
4261	trpC2 Δ*mbl* (Cm)	(5)
BD4893	his, leu-8, metB5 Δ*comK* (Spc)	(6)
168 Δ*upp*	trpC2 *upp*::Pl_{ambda}-*neo*	(7)
ABS1370	trpC2 Δ*mreB* (Cm) *amyE*::Pxyl-*spa-mreB* (Spc)	This study
NC57	trpC2 mcComS (Kan)	This study
NC58	trpC2 *amyE*::P_{comGA}-*comGA-gfp* (Cm)	This study
NC59	trpC2 *comK*::*comK-gfp* (Cm)	This study
NC60	trpC2 *comK*::*comK-gfp* (Cm) mcComS (Kan)	This study
NC66	trpC2 *amyE*::P_{xyl}-*spa-mreB* (Spc) mcComS (Kan) *comK*::*comK-gfp* (Cm)	This study
NC91	trpC2 *amyE*::$P_{mreB123}$-*luc* (Cm) mcComS (Kan)	This study
NC92	trpC2 *amyE*::P_{mreB23}-*luc* (Cm) mcComS (Kan)	This study
NC93	trpC2 *amyE*::P_{mreB3}-*luc* (Cm) mcComS (Kan)	This study
NC94	trpC2 *amyE*::P_{mbl12}-*luc* (Cm) mcComS	This study
NC95	trpC2 *amyE*::P_{mbl2}-*luc* (Cm) mcComS (Kan)	This study
NC96	trpC2 *amyE*::P_{mreBH}-*luc* (Cm) mcComS (Kan)	This study
NC102	trpC2 *upp*::Pl_{ambda}-*neo*, Δ*radC (phleo)*	This study
NC103	trpC2 *upp*::Pl_{ambda}-*neo*, *mreB*::*gfp-mreB*	This study
NC118	trpC2 *thrC*::P_{comGA}-*comGA-mrfpruby* (Ery) mcComS (Phleo)	This study
NC121	trpC2 *mreB*::*gfp-mreB* (Neo) *thrC*::P_{comGA}-*comGA-mrfpruby* (Ery) mcComS (Phleo)	This study
NC122	trpC2 *mbl*::*mbl-gfp* (Cm) *thrC*::P_{comGA}-*comGA-mrfpruby* (Ery) mcComS(Phleo)	This study
NC123	trpC2 *thrC*::P_{comGA}-*comGA-mrfpruby* (Ery) mcComS (Phleo) Δ*mreB* (Cm)	This study
NC129	trpC2 P_{comK}-*luc* (Cm)	This study
NC130	trpC2 P_{comK}-*luc* (Cm) Δ*mreB* (Kan)	This study
NC135	trpC2 *amyE*::P_{xyl}-*perR-spa* (Spc) mcComS (Kan) *comK*::*comK-gfp* (Cm)	This study
NC146	trpC2 *amyE*::$P_{mreB123}$-*luc* (Cm) mcComS (Kan) Δ*comK* (Spc)	This study
NC147	trpC2 *amyE*::P_{mreB23}-*luc* (Cm) mcComS (Kan) Δ*comK* (Spc)	This study
NC148	trpC2 *amyE*::P_{mreB3}-*luc* (Cm) mcComS (Kan) Δ*comK* (Spc)	This study
NC149	trpC2 *amyE*::P_{mbl12}-*luc* (Cm) mcComS (Kan) Δ*comK* (Spc)	This study
NC150	trpC2 *amyE*::P_{mbl2}-*luc* (Cm) mcComS (Kan) Δ*comK* (Spc)	This study
NC151	trpC2 *amyE*::P_{mreBH}-*luc* (Cm) mcComS (Kan) Δ*comK* (Spc)	This study
NC160	trpC2 P_{comK}-luc (Cm) Δ*comK* (Spc)	This study
NC161	trpC2 *comK*::*comK-gfp* (Cm) *ΔmreB* (Kan)	This study
NC162	trpC2 *comK*::comK-*gfp* (Cm) *Δmbl* (Spc)	This study
NC164	trpC2 *comK*::*comK-gfp* (Cm) *ΔcomGA* (Ery)	This study
NC165	trpC2 *comK*::*comK-gfp* (Cm) *ΔmreB* (Kan) mcComS (Phleo)	This study
NC169	trpC2 *comK*::*comK-gfp* (Cm) *ΔmreB* (Kan) *ΔcomGA* (Ery)	This study

NC170	trpC2 *comK*::*comK-gfp* (Cm) *Δmbl* (Spc) *ΔcomGA* (Ery)	This study
NC175	trpC2 P_{comGA}-*luc* (Cm)	This study
NC176	trpC2 P_{comGA}-*luc* (Cm) Δ*mreB* (Kan)	This study
NC197	trpC2 *comK*::*comK-gfp* (Cm) *amyE*::(P_{xyl}-*spa-mreB*) (Spc)	This study
NC203	trpC2 *amyE*::P_{comGA}-*comGA-gfp* (Ery) Δ*mreB* (Kan)	This study
NC208	*trpC2 thrC::*P_{hs}*-comGA-mrfpruby (Ery)*	*This study*
NC215	trpC2 *mreB*::*gfp-mreB* (Neo) *amyE*::P_{comK}-*mrfpruby* (Spc) *ΔcomGA* (Ery)	This study

[(1)] The source of the constructs and strains not created in this study is as follow:

The mcComS and *comK*::*comK-gfp* constructs come from strain BD4015 realized in D. Dubnau's lab.

The *mbl*::*mbl-gfp* construct was first published in strain RCL51 (8).

REFERENCES

1. Albertini AM & Galizzi A (1999) The sequence of the trp operon of Bacillus subtilis 168 (trpC2) revisited. *Microbiology* 145 (Pt 12):3319-3320.
2. Formstone A & Errington J (2005) A magnesium-dependent mreB null mutant: implications for the role of mreB in Bacillus subtilis. *Molecular microbiology* 55(6):1646-1657.
3. Kawai Y, Daniel RA, & Errington J (2009) Regulation of cell wall morphogenesis in Bacillus subtilis by recruitment of PBP1 to the MreB helix. *Molecular microbiology* 71(5):1131-1144.
4. Carballido-López R*, et al.* (2006) Actin homolog MreBH governs cell morphogenesis by localization of the cell wall hydrolase LytE. *Developmental cell* 11(3):399-409.
5. Schirner K & Errington J (2009) The cell wall regulator {sigma}I specifically suppresses the lethal phenotype of mbl mutants in Bacillus subtilis. *Journal of bacteriology* 191(5):1404-1413.
6. Mirouze N, Desai Y, Raj A, & Dubnau D (2012) Spo0A~P imposes a temporal gate for the bimodal expression of competence in Bacillus subtilis. *PLoS genetics* 8(3):e1002586.
7. Fabret C, Ehrlich SD, & Noirot P (2002) A new mutation delivery system for genome-scale approaches in Bacillus subtilis. *Molecular microbiology* 46(1):25-36.
8. Jones LJ, Carballido-Lopez R, & Errington J (2001) Control of cell shape in bacteria: helical, actin-like filaments in Bacillus subtilis. *Cell* 104(6):913-922.
