# Supplementary material for: MreB-Dependent Inhibition of Cell Elongation during the Escape from Competence in Bacillus subtilis
Source: PLoS Genet. 2015 Jun 19;11(6):e1005299. doi: 10.1371/journal.pgen.1005299 (PMC4474612; doi:10.1371/journal.pgen.1005299)
Supplement: S2 Table — (PDF) [file pgen.1005299.s012.pdf]

## S2 Table. Primers used in this study.

| Name | Sequence |
|---|---|
| **Luciferase fusions:** | |
| amyF-F | 5'- AAT TCA TGT AAA AGA TGA GGT TGG TTC ATT CTC GAA AAT T -3' |
| amyR-R | 5'- AAT TCC GGC GAC TGT TTC TGT TTC AGC CT -3' |
| MCS-F | 5'- CGA CTC TAG AGG ATC CCC GGG TAC C -3' |
| MCS-R | 5'- GTG CCA AGC TTG CAT GCC TGC AGG T -3' |
| MCS-mreB1-F | 5'- G TGC CAA GCT TGC ATG CCT GCA GGT ACA GGC AGA ACA AGA GGT TGC GGC -3' |
| MCS-mreB2-F | 5'- G TGC CAA GCT TGC ATG CCT GCA GGT GAC AAA ACC GCT AAT ACT TGC ATC ACA ATC TCC G -3' |
| MCS-mreB3-F | 5'- G TGC CAA GCT TGC ATG CCT GCA GGT GGT CAT ACA CGA TCT GCC ATT AAA ACT CAA AGA TTT CC -3' |
| RBS-mreB-R | 5'- G GTA CCC GGG GAT CCT CTA GAG TCG GCA AAA ATA CCC TAA AGG GAA AAA CTC ATA AAC TCT ATT ATA GC -3' |
| MCS-mbl1-F | 5'- G TGC CAA GCT TGC ATG CCT GCA GGT CGC TCA ACG ATG GCT CCG GAG AAA -3' |
| MCS-mbl2-F | 5'- G TGC CAA GCT TGC ATG CCT GCA GGT GCA CGA TTA CAT CAA AGA GCG AAC AAT CAA GAT AGG -3' |
| RBS-mbl-R | 5'- G GTA CCC GGG GAT CCT CTA GAG TCG GAA AAT CTG TTT CAC AGT AAA TAT CCA CAT TGT GCC C -3' |
| MCS-mreBH-F | 5'- G TGC CAA GCT TGC ATG CCT GCA GGT GCG GTT TTT TTC TTC AGA AAG CTG TTT ATA CTT CCA -3' |
| RBS- mreBH-R | 5'- G GTA CCC GGG GAT CCT CTA GAG TCG ATC CTA ATT TAA TAT GAT TCT ACA TTT AAA GTT TCT CAG AAA TAC TGC GA -3' |
| RBS- mreBH-R | 5'- G GTA CCC GGG GAT CCT CTA GAG TCG ATC CTA ATT TAA TAT GAT TCT ACA TTT AAA GTT TCT CAG AAA TAC TGC GA -3' |
| **SPA fusion:** | |
| **Pxyl-spa-mreB** | |
| ac983 | 5'- CTC CTC GAG ATG TTT GGA ATT GGT GCT AGA G -3' |
| ac984 | 5'- GAA GAA TTC ACC TCT TCT ATT GAA CTC CCG -3' |
| **FP fusions:** | |
| **$P_{native}$-GFP-mreB** | |
| ∗ ΔradC: | |
| HindIII-Pmaf-F | 5'- GCA GCA AAG CTT GGA AAG ACC GGG GCT GCC AAA CCG -3' |
| Phleo-radC-R | 5'- CGA CCT GCA GGC ATG CAA GCT CCA AGC ACT CCT TTT CTT TCC CTT CCC G -3' |
| K7PH_F | 5'- AGC TTG CAT GCC TGC AGG TCG AC -3' |
| K7PH_R | 5'- CGA CGG CCA GTG AAT TCG AGC TC -3' |
| Phleo-radC-F | 5'- CGA GCT CGA ATT CAC TGG CCG TCG GGC TGT TTG AAT GCG GAA ACC TGA TTG -3' |
| HindIII-mreB-R | 5'- GCA GCA AAG CTT TTA TCT AGT TTT CCC TTT GAA AAG ATG GAT GTG CTC C -3' |
| ∗ Pmaf-maf-radC-GFP-mreB: | |
| Pmaf-F | 5'- GGA AAG ACC GGG GCT GCC AAA CCG -3' |
| GFP-radC-R | 5'- ACT CAT CTG CAG GGT ACC CAT ATG TAT GTA TCT TCC TTT CTT AAA GCA AAA ATA CCC TAA AGG G -3' |

| | |
|---|---|
| RBSmreB-GFP-F | 5'- GGG TAT TTT TGC TTT AAG AAA GGA AGA TAC ATA CAT ATG GGT ACC CTG CAG ATG AGT AAA GG -3' |
| mreB-R | 5'- TTA TCT AGT TTT CCC TTT GAA AAG ATG GAT GTG CTC C -3' |

**$P_{native}$-*comGA-RFP* :**

| | |
|---|---|
| hom-F | 5'- AAT TCA TGT AAA AGA TGA GGT TGG TTC ATT CTC GAA AAT T -3' |
| pDG1664-MCS-R | 5'- GAT AAG CTT CTA GGA TCC GAA GGC AGC A -3' |
| pDG1664-MCS-PcomGA-F | 5'- TGC TGC CTT CGG ATC CTA GAA GCT TAT C CTC GGC GAA CTA ATG AAT GCG TAT TTA ATC GG -3' |
| RFP-comGA-R | 5'- GCT GGC CAT GGT AAG CTT GCC CAT ATC TTT TTC ATG ATA AAC CCA CCG GTC ATA GTT GTT TGT CGT -3' |
| comGA-RFP-F | 5'- ACG ACA AAC AAC TAT GAC CGG TGG GTT TAT CAT GAA AAA GAT ATG GGC AAG CTT ACC ATG GCC AGC -3' |
| pDG1664-MCS-RFP-R | 5'- TCT TAA GTT TGC TTC TAA GTC TTA TTT CCA TAA CTT TAG GGT TAT C TTA AGC GCC TGT GCT ATG TCT GCC CTC -3' |
| pDG1664-MCS-F | 5'- GAT AAC CCT AAA GTT ATG GAA ATA AGA CTT AGA AGC AAA CTT AAG A-3' |
| thrB-R | 5'- AAT TCC GGC GAC TGT TTC TGT TTC AGC CT -3' |

**$P_{hyperspank}$-*comGA-RFP* :**

| | |
|---|---|
| hom-F | 5'- AAT TCA TGT AAA AGA TGA GGT TGG TTC ATT CTC GAA AAT T -3' |
| pDG1664-MCS-R | 5'- GAT AAG CTT CTA GGA TCC GAA GGC AGC A -3' |
| pDG1664-MCS-comGA-F | 5'- CCT CGT TTC CAC CGA ATT AGC TTG CAT GC CTA ATC TTT TTC ATG ATA AAC CCA CCG GTC ATA GTT GTT-3' |
| RFP-comGA-R | 5'- GCT GGC CAT GGT AAG CTT GCC CAT ATC TTT TTC ATG ATA AAC CCA CCG GTC ATA GTT GTT TGT CGT -3' |
| comGA-RFP-F | 5'- ACG ACA AAC AAC TAT GAC CGG TGG GTT TAT CAT GAA AAA GAT ATG GGC AAG CTT ACC ATG GCC AGC -3' |
| pDG1664-MCS-RFP-R | 5'- TCT TAA GTT TGC TTC TAA GTC TTA TTT CCA TAA CTT TAG GGT TAT C TTA AGC GCC TGT GCT ATG TCT GCC CTC -3' |
| pDG1664-MCS-F | 5'- GAT AAC CCT AAA GTT ATG GAA ATA AGA CTT AGA AGC AAA CTT AAG A-3' |
| thrB-R | 5'- AAT TCC GGC GAC TGT TTC TGT TTC AGC CT -3' |

**$P_{comK}$-*RFP* :**

| | |
|---|---|
| amyF-F | 5'- AAT TCA TGT AAA AGA TGA GGT TGG TTC ATT CTC GAA AAT T -3' |
| amyR-R | 5'- AAT TCC GGC GAC TGT TTC TGT TTC AGC CT -3' |
| PcomK-amyF-R | 5'- GGT TCT GAT CAG CTG GTG GCT GGT G GCG TGA CAT CCC ATC GAT CAG ACC AG -3' |
| amyF-PcomK-F | 5'- CTG GTC TGA TCG ATG GGA TGT CAC GC CAC CAG CCA CCA GCT GAT CAG AAC C -3' |
| RFP-PcomK-R | 5'- CCT CGG AGC TGG CCA TGG TAA GCT TGC CCA T ATT ATG GCC TCC ATC CTT TTT CTG CAA AAT TTA TAC TAA T -3' |
| PcomK-RFP-F | 5'- ATT AGT ATA AAT TTT GCA GAA AAA GGA TGG AGG CCA TAA T ATG GGC AAG CTT ACC ATG GCC AGC TCC GAG G -3' |
| amyR-RFP-R | 5'- TCG CCA TTC GCC AGG GCT GCA GGA ATT C TTA AGC GCC TGT GCT ATG TCT GCC CTC AG -3' |
| RFP-amyR-F | 5'- CTG AGG GCA GAC ATA GCA CAG GCG CTT AA GAA TTC CTG CAG CCC TGG CGA ATG GCG A -3' |
