# Supplementary material for: MreB-Dependent Inhibition of Cell Elongation during the Escape from Competence in Bacillus subtilis
Source: PLoS Genet. 2015 Jun 19;11(6):e1005299. doi: 10.1371/journal.pgen.1005299 (PMC4474612; doi:10.1371/journal.pgen.1005299)
Supplement: S3 Table — Table showing the mean length of competent and non-competent cells at T2 and the mean length of competent cells at T2+90 minutes. All the strains presented here carry a ComK-GFP fusion in order to differentiate the competent cells from the non-competent. Lengths are given in micrometers. The numbers in parentheses are standard deviations. No fewer than 100 competent cells were measured for each data point. ND stands for Not Determined. In the last column is presented a statistical test (Student’s paired t-test) revealing how significant is the size difference observed between competent cell of the wild-type and the other strains at T2+90. By conventional criteria, a two-tailed P value inferior to 0,0001 is considered to be extremely statistically significant. In contrast all the other P values are considered to be not quite statistically significant. (PDF) [file pgen.1005299.s013.pdf]

S3 Table. Cell length during outgrowth.

Genotype	T2 Comp	T2 Non-comp.	T2 + 90min Comp.	Paired *t*-test P value
wild-type (NC59)	2,83 (0,57)	2,90 (0,62)	3,88 (1,03)	-
ΔcomGA (NC164)	2,84 (0,57)	2,91 (0,66)	11,61 (4,06)	< 0,0001
ΔmreB (NC161)	2,41 (0,53)	2,49 (0,45)	3,39 (0,81)	< 0,0001
ΔcomGA, ΔmreB (NC169)	2,43 (0,65)	2,48 (0,74)	3,6 (1,4)	0,0546
ΔcomGA, ΔmreB 25 mM Mg^{2+} (NC169)	ND	ND	3,92 (1,16)	0,8257
Δmbl (NC162)	2.74 (0.88)	2,81 (0,99)	3,95 (1,17)	0,6274
ΔcomGA, Δmbl (NC170)	2.54 (0.68)	2,65 (0,72)	7,27 (2,34)	< 0,0001
ΔcomGA, Δmbl 25 mM Mg^{2+} (NC170)	ND	ND	10,31 (3.4)	< 0,0001
amyE::P_{xyl}*-spa-mreB* (NC197)	2.60 (0.64)	2,67 (0,75)	6,31 (3,35)	< 0,0001

Table showing the mean length of competent and non-competent cells at T_2 and the mean length of competent cells at T_2+90 minutes. All the strains presented here carry a ComK-GFP fusion in order to differentiate the competent cells from the non-competent. Lengths are given in micrometers. The numbers in parentheses are standard deviations. No fewer than 100 competent cells were measured for each data point. ND stands for Not Determined.

In the last column is presented a statistical test (Student's paired t-test) revealing how significant is the size difference observed between competent cell of the wild-type and the other strains at T2+90. By conventional criteria, a two-tailed P value inferior to 0,0001 is considered to be extremely statistically significant. In contrast all the other P values are considered to be not quite statistically significant.
